# A Reporter System for Assessment of Transcription from Divergently Oriented Promoters in *Pseudomonas putida*

**DOI:** 10.1021/acssynbio.5c00723

**Published:** 2025-12-10

**Authors:** Johanna Hendrikson, Mia-Lota Keskküla, Gea M. Räis, Maia Kivisaar, Riho Teras

**Affiliations:** Institute of Molecular and Cell Biology, 37546 University of Tartu, Tartu 51010, Estonia

**Keywords:** *Pseudomonas putida*, reporter vector, Scarlet-I3, SYFP2, excludon, divergently orientated promoters

## Abstract

*Pseudomonas putida* is a metabolically versatile bacterium widely used in industrial biotechnology and synthetic biology. However, the lack of rapid, sensitive, and noninvasive tools for monitoring gene expression in *P. putida* limits the opportunities to study its gene regulation. We developed a plasmid-based dual-reporter system optimized for *P. putida*, which enables simultaneous monitoring of gene expression from promoter areas that contain divergently orientated promoters. Two fluorescent proteins (SYFP2 and Scarlet-I3) were selected for a reporter based on their compatibility with the intrinsic autofluorescence of *P. putida* and their detectability in LB medium. We engineered plasmid backbones containing the BBR1 and RK2 origins of replication and incorporated the toxin-antitoxin module *hok-sok* to ensure plasmid maintenance without antibiotic selection, making it possible to use this system to quantify gene expression in both planktonic and sessile (biofilm) states. Additionally, we created reporter systems with fused reporter genes with protein half-life decreasing tags, allowing dynamic assessment of transcriptional activity. Using confocal microscopy, we demonstrated spatially distinct expression patterns of biofilm-related genes (e.g., *lapF*) within mature biofilms. We also tested excludon-based transcriptional repression of a reporter gene in *P. putida* using this system, but observed limited efficiency under the tested conditions.

## Graphical abstract

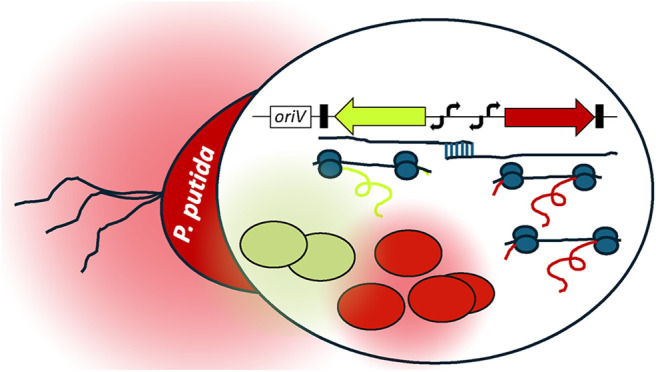

## Introduction

*Pseudomonas putida* is a Gram-negative bacterium characterized by its broad metabolic versatility and ease of genetic manipulation, making it a valuable host for industrial applications. It has a high solvent tolerance and an ability to tolerate various environmental conditions. As a result, this bacterium is widely used for biotechnological and synthetic biology applications. The most common strains of *P. putida* used in research are KT2440 and its isogenic strain PaW85, which are plasmid-free. Both were derived independently from *P. putida* mt-2, which contains the TOL plasmid pWW0. KT2440 has been extensively studied and is considered a biosafety level 1 organism for recombinant DNA work. The main focus of extensive characterization has been on *P. putida* KT2440; however, the isogenic strain PaW85 shares the same genetic background and thus the knowledge gained from KT2440 is largely applicable to PaW85. Although *P. putida* strains have been studied extensively, there is an issue with the lack of genetic tools capable of precise and real-time monitoring of gene expression, especially under conditions relevant to industrial and environmental processes, such as biofilm formation or growth in complex environments.^1–4^

Fluorescent protein (FP)-based reporter systems are widely used for noninvasive monitoring of transcriptional and translational activity in microbial systems. However, the autofluorescence of pseudomonads, especially in the green spectral range, and the fluorescence of components in complex media such as LB, limit the range of FPs which can be used to accurately measure changes in gene expression. Often, the common practice has been to use the same FPs in both *E. coli* and *P. putida*, however, the potential interference from medium components and the organism’s autofluorescence has not been systematically assessed. Furthermore, the stable FPs accumulation can mask changes in gene expression, making assessing promoter strength, transcriptional regulation, and stress responses problematic.^5,6^

The primary goal of this study was to construct a reporter plasmid allowing for the study of bidirectional transcription. Therefore, we had to identify a pair of FPs suitable for universal application in gene expression studies in *P. putida*. Special attention was given to complex media, with Lysogeny Broth (LB) medium selected as the representative example. Although defined minimal media, such as M9, are widely used in *P. putida* research, they are not always appropriate for investigating bacterial physiology in more natural or applied contexts.

M9 medium contains an unusually high concentration of inorganic phosphate (approximately 42 mM), which is rarely encountered in natural environments (typically less than 10 μM, depending on soil type).^7–9^ Abundant or excessive phosphate concentrations may significantly alter bacterial physiology, potentially masking key regulatory responses and distorting the output of transcriptional reporters. Additionally, a growth medium’s peptidic component is critical for initiating biofilm formation in *P. putida* PaW85.^10,11^ These reasons indicate that using M9 medium might hinder the detection of certain *P. putida* phenotypes. Complex macromolecules in the growth environment, such as peptides, DNA, fatty acids, and polysaccharides, can modulate gene expression in ecologically and physiologically relevant ways. Given that *P. putida* is a rhizosphere-dwelling bacterium, it is reasonable to assume that it has adapted to environments rich in macromolecular debris released from plant root epidermal cells during senescence.^12,13^ Consequently, LB medium containing yeast extract and peptides was chosen as a proxy for a macromolecule-rich environment.

Given the compact organization of bacterial genomes, wherein genes are often adjacent and oriented in opposite directions, it is possible (and perhaps even preferred) for cells to control the expression of such genes with shared promoter regions concurrently. This kind of regulation, particularly involving oppositely oriented promoters, may result from RNA polymerase (RNAP) competition, collision events between RNAPs (or RNAP and DNAP), or excludon-mediated processes. All these mechanisms require a case-by-case study rather than mass analyses, particularly when the mentioned mechanism affects both transcription and translation. Therefore, gene expression is examined by comparing the native promoter area with the promoter area where one-directional promoters are mutated, revealing the true potential of other-directional promoters. Since gene expression encompasses both transcription and translation, it is necessary to develop a reporter system that can easily assess both stages. Furthermore, modulating bacterial gene expression using excludon-mediated sensing mechanisms has potential uses in industrial applications or experimental settings, where fine-tuning gene expression levels is necessary.^14–16^

In this study, we constructed a reporter system consisting of two FP genes optimized for use in *P. putida* to overcome these limitations. The reporter system was designed to allow simultaneous monitoring of bidirectional transcription, transcriptional activation, or repression from promoters under interest and to function under nonselective conditions, such as in mature biofilms, where antibiotic diffusion is limited.

We selected FPs with minimal spectral overlap and low background signal in *P. putida* and LB media. The half-life of the reporter protein was reduced using C-terminal tag peptides to improve the temporal resolution of expression dynamics. In addition, we investigated the possibility of excludon-based regulation, a mechanism described initially in *Listeria monocytogenes* that involves the excludon of overlapping sense and antisense transcription, as a novel means of controlling gene expression in *P. putida*.^17,18^ Although our results showed limited repression efficiency in this organism, the architecture of the reporter system we developed is readily adaptable to test other regulatory mechanisms. Our results display a versatile and sensitive reporter platform adapted for *P. putida*, which enables detailed gene expression analysis in both planktonic and biofilm growth, in rich and minimal environments. The developed reporter platform could also be used in *E. coli*, as these plasmids replicate in *E. coli*, and the emission of the fluorescent proteins used in this study can be detected from *E. coli*. This work lays a foundation for further studies on transcriptional regulation and synthetic control systems in *P. putida*, supporting its application in sustainable bioproduction and environmental biotechnology.

## Results

### Identification of Optimal Fluorescent Proteins

To investigate gene expression, we opted for a fluorescence-based reporter system due to several advantages: (i) minimal invasiveness, since fluorescence measurements do not require cell lysis, unlike β-galactosidase assays or RT-qPCR-based methods; (ii) speed and efficiency, fluorescence assays require significantly less labor than other indirect gene expression analysis techniques; (iii) real-time monitoring, gene expression can be assessed repeatedly at short intervals, which is not feasible with invasive methods. Our model organism, *P. putida*, exhibits intrinsic fluorescence.^19,20^ Thus, our first objective was to identify two fluorescent proteins (FPs) with emission spectra distinct from the bacterium’s background fluorescence in defined M9 and LB media. Furthermore, as the goal was to construct a bidirectional reporter system, we required two FPs with specific properties: (i) monomeric structure to prevent oligomerization artifacts; (ii) similar and rapid maturation times to ensure synchronized fluorescence development; (iii) comparable brightness to enable accurate relative quantification of gene expression. Therefore, we selected four monomeric FPs for initial experiments: TagBFP (Em 457 nm, considered as blue fluorescence), mCerulean (Em 475 nm, cyan), mVenus (Em 527, yellow), Scarlet-I (Em 593 nm, red) and a standard FP, Gfpmut2 (Em 508, green, a weak dimer; Table S1).^21–26^ Later, when the new Scarlet-I variant Scalet-I3 was published, we added Scarlet-I3 (Em 592 nm, red) and SYFP2 (Em 527 nm, yellow, analogue to mVenus) to the study as a new pair of FP with similarly short maturation times, respectively 2.0 and 4.1 min (Table S1).^24,26,27^

The objective was to identify emission wavelengths at which the medium components and *P. putida* produce minimal autofluorescence, < 100 fluorescence units (FU). Fluorescence values above 100 FU or elevated levels of cells’ autofluorescence (compared to the media) would hinder further fluorescence-based measurement of gene expression. Of all media, the M9 0.2% (w/v) glucose exhibited the lowest background fluorescence (Figure 1A). However, the cellular autofluorescence in the M9 0.2% (w/v) glucose still interfered with fluorescence detection at the excitation and emission wavelengths of TagBFP (BFP) and mCerulean (CFP), because the background fluorescence of cells in M9 0.2% (w/v) glucose medium was higher than 1000 FU (Figure 1A). The cells’ autofluorescence did not differ from the medium’s fluorescence at the excitation and emission wavelengths of Gfpmut2, mVenus (SYFP2) or mScarlet-I (mScarlet-I3), suggesting that the cells do not emit light at these wavelengths (Figure 1A). Therefore, M9 0.2% (w/v) glucose and M9 0.2% (w/v) glucose 0.2% (w/v) CAA were suitable for fluorescence measurements at excitation and emission wavelengths above 488 nm (±20 nm) and 510 nm (±20 nm), respectively, covering the spectral ranges of Gfpmut2 (GFP), mVenus and Scarlet-I (Figure 1A).

**Figure 1:**
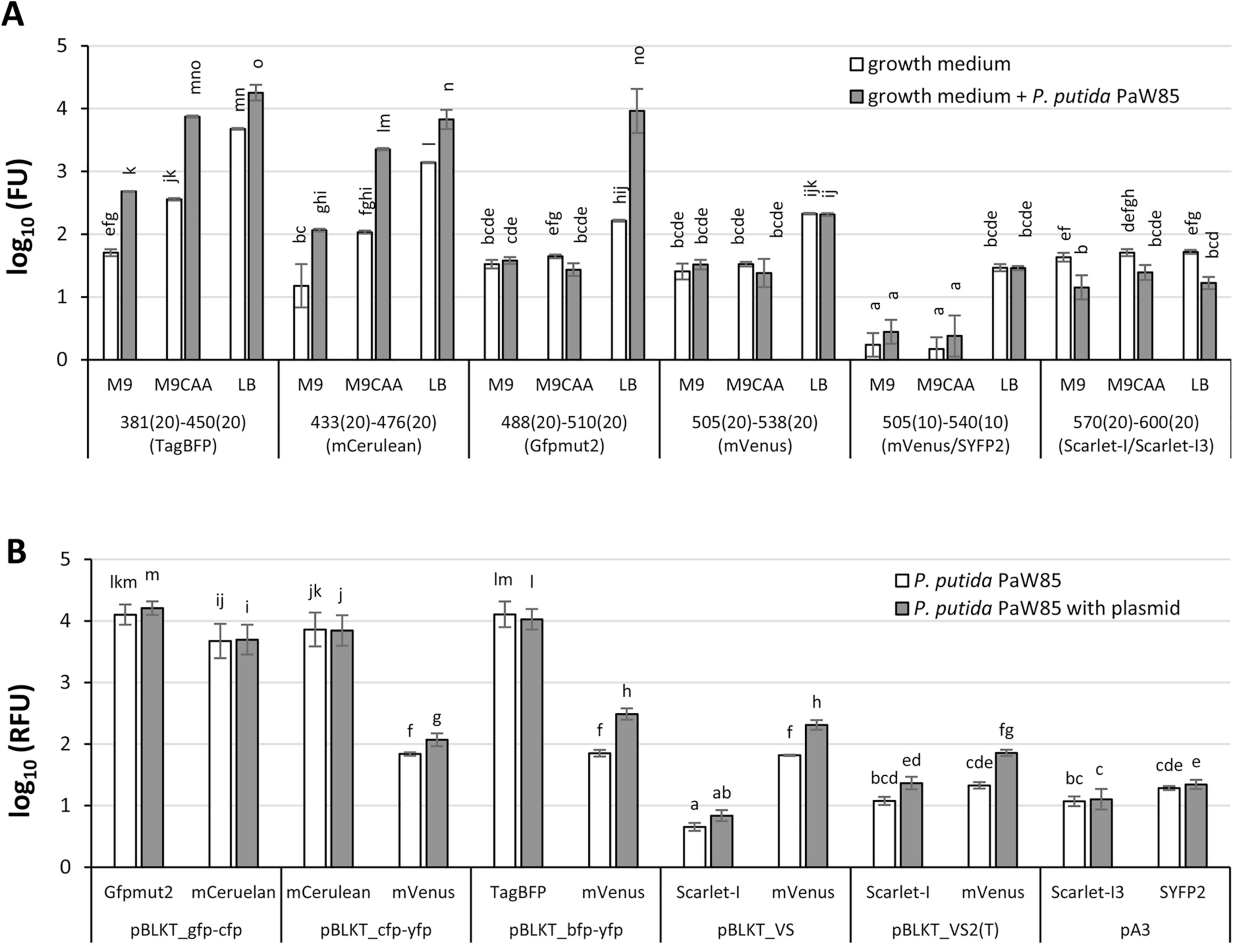
Background fluorescence of growth media and *P. putida* PaW85 and the relative fluorescence (RFU) of *P. putida* PaW85 cells, both plasmid-free and harboring various reporter plasmids. (A) Fluorescence measurements were performed for fresh M9 0.2% (w/v) glucose (denoted as M9), M9 0.2% (w/v) glucose +0.2% (w/v) CAA + tryptophan (denoted as M9CAA), and LB medium, as well as for plasmidless *P. putida* PaW85 grown in these media. (B) The relative fluorescence (RFU) of plasmid-free *P. putida* PaW85 and cells harboring empty reporter plasmids. Bacteria were grown for 18 h at 30 °C, 180 rpm. Measurements were taken at the excitation and emission wavelengths corresponding to potential fluorescent reporter proteins: TagBFP (BFP): Ex 381 nm (±20), Em 450 nm (±20), mCerulean (CFP): Ex 433 nm (±20), Em 476 nm (±20), Gfpmut2 (GFP): Ex 488 nm (±20), Em 510 nm (±20), mVenus in pBLKT_VS: Ex 505 nm (±20), Em 538 nm (±20), mVenus in pBLKT_VS2­(T) and SYFP2 in pA3: Ex 505 nm (±10), Em 540 nm (±10), Scarlet-I and Scarlet-I3: Ex 570 nm (±20), Em 600 nm (±20). Displayed values represent the arithmetic mean, with error bars indicating the 95% confidence interval. Multifactorial ANOVA was calculated from log10 values, with homogeneity groups indicated above the bars (*n* = 9).

In contrast, the displayed fluorescence intensity of LB medium was inversely proportional to wavelength, with background fluorescence decreasing as emission wavelengths increase (Figure 1A). Additionally, *P. putida* exhibited significant intrinsic fluorescence, particularly below 530 nm, making fluorescence measurements at shorter wavelengths unreliable in LB-grown cultures (Figure 1A). At 510 nm (±20 nm), LB-grown *P. putida* exhibited fluorescence levels ∼200-fold higher than LB alone. However, in M9 0.2% (w/v) glucose and M9CAA, fluorescence at 510 nm (±20 nm) remained low, ranging from 28 to 44 FU, regardless of the presence of cells (Figure 1A).

A critical threshold for mVenus measurements was observed at 530 nm, where LB background fluorescence decreased from 210 FU (538 nm ± 20 nm) to 29 FU (540 nm ± 10 nm) by narrowing the bandwidth from ± 20 nm to ± 10 nm (Figure 1A). In the red spectral range (Ex 570 nm (±20)/Em 600 nm (±20)), fluorescence from the medium + cells was below 25 FU, 2–3 times lower than that of the medium alone (Figure 1A). This suggests that bacteria degrade medium components or *P. putida* absorbs red light to some extent.

In sum, we assessed the emission of light of three media: M9 0.2% (w/v) glucose, M9 0.2% (w/v) glucose +0.2% (w/v) CAA + tryptophan and LB, with and without plasmidless *P. putida*; and ascertained that the background fluorescence of *P. putida* and complex media components (such as LB) is minimal above 530 nm, which means the YFP and RFP proteins, such as mVenus (as well as SYFP2) and Scarlet-I (as well as Scarlet-I3; Table S1, Figure 1A), should be suitable for a *P. putida* reporter system.

### Assessment of Potential Promoters in Front of Reporter Genes

To create a bidirectional reporter system, we had to assess whether the selected fluorescent reporter genes contain promoters within their coding sequence. We had already constructed pBLKT_gfp-cfp, pBLKT_cfp-yfp and pBLKT_bfp-yfp containing two divergently orientated reporter genes forming a reporter cassette (Table S3). We assessed the autofluorescence of cells carrying so-called empty plasmids (which did not contain promoters between the two reporter genes). The autofluorescence of cells carrying plasmids was higher than that of plasmid-free cells only in the case of at the wavelength of YFP (mVenus; Figure 1B). Since the reporter cassette was flanked by transcriptional terminators, we controlled the idea that reporter genes themselves may contain promoters that could cause expression of the reporter gene in the opposite direction. Therefore, these promoters may hinder the usability of the reporter vector. To address this issue, *in silico* promoter predictions were performed within the reporter gene cassette (Table S2). Thus, considering that *P. putida*’s and the growth medium’s background fluorescence was low only at wavelengths above 530 nm (Figure 1A), the following study was focused on YFP and RFP-s. From now on, all subsequent mVenus and SYFP2 emission measurements were performed with the narrow bandwidth (540 nm ± 10 nm). Based on these *in silico* predictions, synthetic promoter-free DNA sequences (stDNA; Twist Bioscience HQ, San Francisco) were designed to construct three generations of reporter systems utilizing yellow and red FPs: (i) modified DNA containing mVenus and Scarlet-I genes in plasmid pBLKT-VS, (ii) previously published sequence containing mVenus^28^ and Scarlet-I^29^ genes in pBLKT-VS2­(T), and (iii) modified DNA containing SYFP2 and Scarlet-I3 genes in BBR1 origin plasmids pA/pB, and in RK2 origin plasmid pYR/pRY (the differences of the third generation plasmids are commented later; Tables S1, S2 and S3).

The brightness and maturation times for the proteins selected for constructing a working reporter plasmid had to be as similar as possible. The two proteins having comparable maturation times ensure that the two genes’ expression can be assessed precisely and simultaneously, without causing an illusory shift in the time of regulation events. Therefore, the pair of mVenus and Scarlet-I (maturation times of 17.6 and 36.0 min, respectively) was chosen for the first and second generation of plasmids due to their relatively high brightness and maturation times (Tables S1 and S3). During the research work, Scarlet-I3 ^27^ was published, which had a significantly shorter maturation time (2.0 min) than its predecessor, Scarlet-I, and therefore, Scarlet-I3 and SYFP2 ^24^ was selected as a pair with a similar maturation time (2.0 and 4.1 min, respectively) for the third generation of plasmids (Tables S1 and S3).

In fact, the fluorescence of plasmid-free cells and cells carrying third-generation plasmids was similar (Figures 1B and S1). This indicates that the opposite-oriented reporter genes did not contain additional promoters, and the transcription terminators flanking the reporter genes were sufficiently effective in excluding interfering transcription from the promoters induced in the plasmid outside of the reporter cassette (Figure 1B and S1).

In conclusion, the third-generation reporter system, based on SYFP2 and Scarlet-I3 with optimized DNA sequences, effectively eliminates unintended expression within the reporter cassette and from the plasmid. The plasmid series names are pA, pB, pYR and pRY. This system provides a robust platform for studying gene regulatory mechanisms in *P. putida*.

### The Selection of the Plasmid Backbone (*oriV*) and Other DNA Elements, Which Were Necessary to Create the Reporter System

Creating both low-copy and medium-copy number versions of the plasmids for the reporter system was necessary to ensure the possibility of assessing the expression of weak and strong promoters, respectively. The BBR1, as a representative of medium-copy-number, and RK2, as a representative of low-copy-number *oriVs*, were selected because they have been widely used in promoter-probe vectors.^30–32^

At first, we assessed the plasmid burden on the cells by the ability to form colonies. Since the growth curve describes the subpopulation of cells that grow fastest (growth rate or generation time is described by a geometric function), colony formation reveals, in addition to the burden of the plasmid on cells, the heterogeneous growth at the single cell level of the population that may be caused by the plasmid. Namely, the larger the colony area, the lower the plasmid burden on the cells. Therefore, the area of colonies grown on LB supplemented with antibiotics (penicillin G and kanamycin) plates for 24 h was assessed (Figure 2A). The heterogeneity of colony area (the impact of the plasmid on colony formation) can be assessed by comparing the mean of colony area and the standard deviation. Particularly high standard deviation compared to the arithmetical mean reflects the fluctuation of data (colony area). Larger fluctuations in the colony area were observed for the pSEVA235 (mean 229 ± SD 177) and pSEVA225 (217 ± SD 110) compared to pBLKT (1033 ± SD 261), pSEVA132 (1231 ± SD 233) or pPR9TT (805 ± SD 153). Additionally, we did not see the clear dependency of plasmid burden on the cell from *oriV* or antibiotic resistance marker, plasmid size or other plasmidial components (Figure 2A). Thus, it seems that the plasmid construction itself may cause an unwanted burden on cells. As our purpose was to find the plasmid that does not burden cells or cause remarkable heterogeneity that would hinder gene expression analyses by different methods, including single-cell analysis, we selected the pBLKT plasmid (BBR1 *oriV*) and the pPR9TTBlacZ plasmid (RK2 *oriV*) for further experimentation. While the antibiotic resistance marker of the plasmids did not play a crucial role in selecting the fluorescent protein pair, for subsequent experiments, to study the maintenance of plasmids in cells, we chose the β-lactamase gene (*bla*) as a selection marker, which does not directly interfere with transcription or translation. This choice ensured that antibiotics do not interfere or have minimal effect on gene expression or excludon assessment (Table S3).

**Figure 2:**
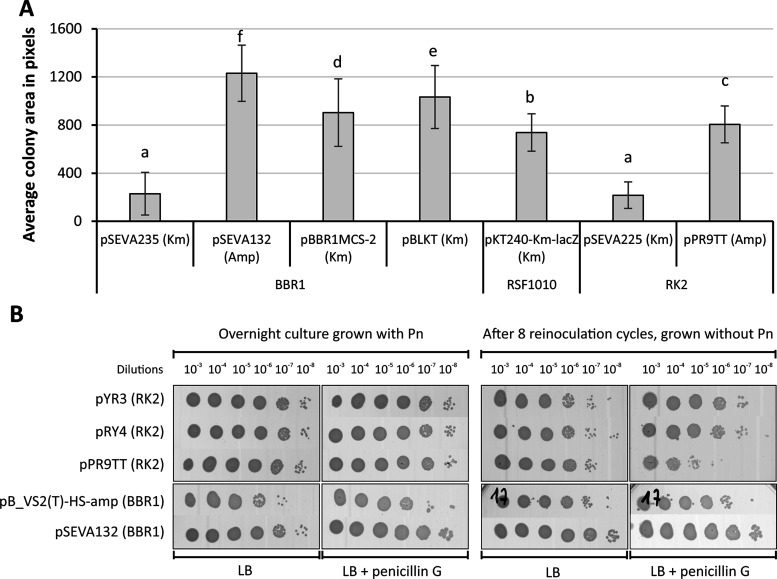
Plasmids’ burden on *P. putida* PaW85 and maintenance of plasmid in cells. (A) The plasmids’ burden is shown by the arithmetic mean of relative colony area (in pixels) grown for 24 h on plates with antibiotics, with error bars indicating the standard deviation. The plasmids with *oriV* BBR1, RSF1010, and RK2 were grouped. Statistical analysis was performed using one-way ANOVA, with homogeneity groups indicated above the columns (*n* ≥ 3). (B) The maintenance of plasmids in cells. Plasmid-containing *P. putida* PaW85 was cultured for 24 h in LB liquid medium supplemented with 1000 μg/mL penicillin G (Pn). The culture was then diluted 50-fold into LB without Pn and incubated for 6–8 h at 30 °C with shaking at 180 rpm. This dilution process was repeated eight times (approximately 45 generations in total with all 8 reinoculations), followed by overnight incubation at 30 °C and 180 rpm. Thereafter, decimal dilutions from overnight cultures were made, and 3 μL of each dilution was plated onto LB and LB + Pn agar plates, and the plates were incubated at 30 °C for 24 h. The experiments with the most significant difference in colony growth between LB and LB + Pn agar plates are represented.

To develop a universal reporter system, we incorporated the toxin-antitoxin system *hok-sok* (Table S3) into the plasmid construct. This system allows the plasmid to be maintained in bacterial cells even without antibiotics, which is particularly useful for studying gene expression in biofilms where antibiotic diffusion is limited. We compared the persistence of plasmids without the *hok-sok* system (pPR9TT and pSEVA132) to those with the *hok-sok* system (pYR3, pRY4, and pB-VS2­(T)-HS, Figure 2B). Surprisingly, plasmids with the BBR1 origin remained stable in the cells for approximately 45 generations even without the *hok-sok* system (colony numbers were similar on both LB and LB+Pn plates; Figure 2B). However, for RK2 plasmids, the *hok-sok* system was essential for ensuring plasmid persistence in all cells (Figure 2B).

### Evaluation of the Created Reporter Plasmids

Since the BBR1 and RK2 constructed plasmids had different terminators surrounding the reporter cassette (Table S2), we next examined whether the background fluorescence of cells with plasmids based on pBLKT (pA3 and pB4) and pPR9TT (pYR3 and pRY4; Table S3, Figure 3) was comparable to that of cells without plasmids.

**Figure 3:**
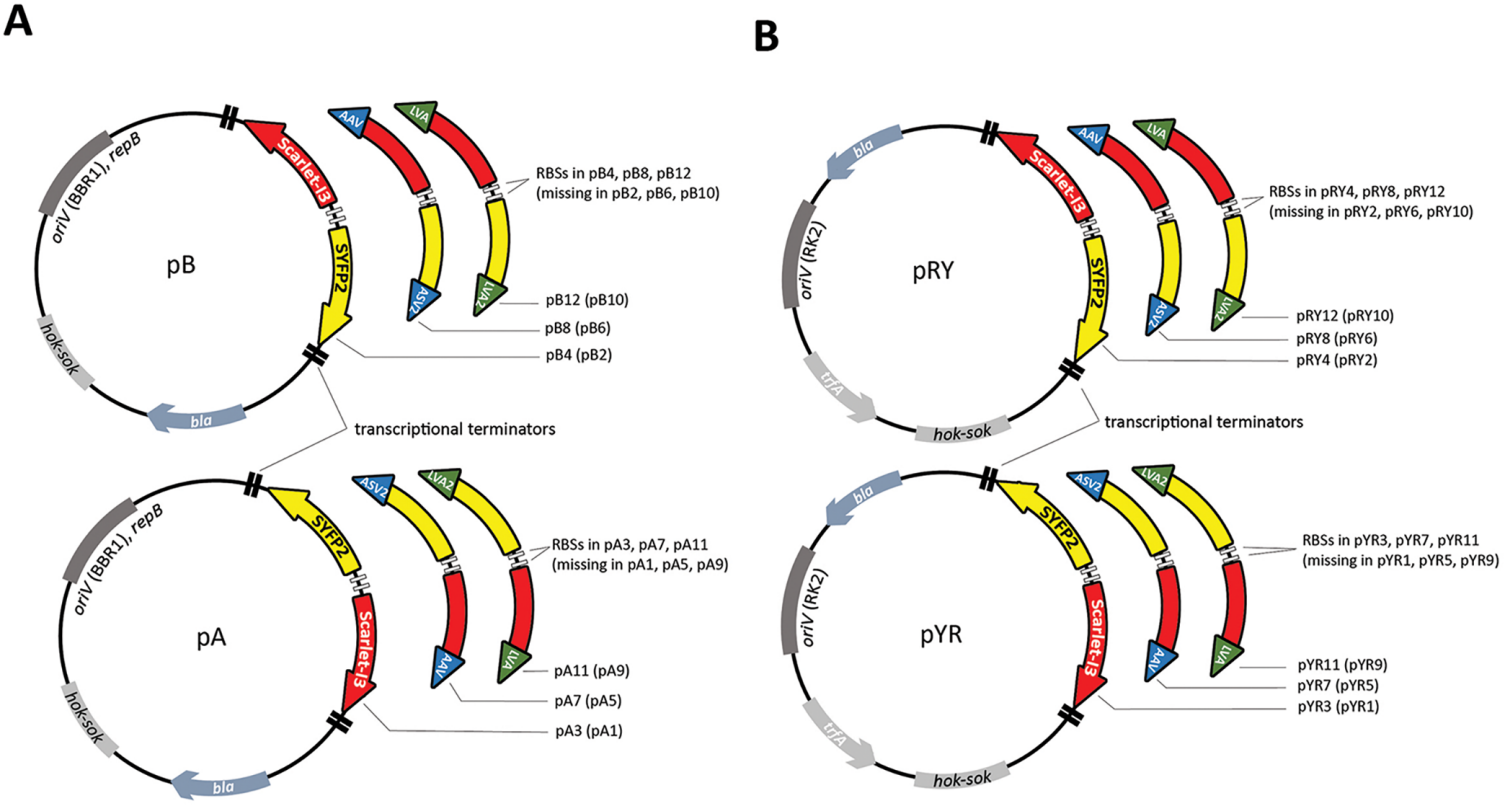
Schematics of constructed plasmids. The left panel (A) shows plasmids with the BBR1 origin (pB and pA), while the right panel (B) shows the plasmids with the RK2 origin (pRY and pYR). The Scarlet-I3 and SYFP2 genes, forming a reporter cassette, are flanked by transcription terminators (small black boxes). pB (pRY) differs from pA (pYR) in the orientation of the reporter cassette. RBSs are indicated as white boxes between the genes, e.g., pB plasmids have RBSs on reporter plasmids pB4, pB8, pB12, but are absent on pB2, pB6, and pB10. In plasmids without RBS, the reporter genes also lack the ATG codon to allow the construction of hybrid proteins with the reporter and target protein genes. Green and blue arrows indicate the sequence of protein half-life shortening tags LVA and LVA2 (e.g., on plasmids pB12 and pB10), AAV and ASV tags (e.g., on plasmids pB8 and pB6). Plasmids without colored arrows, e.g., on pB4 and pB2, have no tags. The β-lactams resistance-determining gene *bla*, *oriV*, and the toxin-antitoxin cassette *hok-sok* are indicated in the diagram. For cloning the promoter region, there is a *Bam*HI sequence between the reporter genes (between the RBSs), which is not shown in the schemes. The plasmids are described in more detail in Table S3.

Fluorescence measurements revealed no significant difference (*p* > 0.486) between plasmid-free cells and those harboring the empty plasmids (Figure S1). This indicates that the promoters in plasmids localized outside the reporter cassette did not interfere with the reporter’s gene expression. Therefore, it is equally highly unlikely that transcription initiated from the promoter cloned into the reporter cassette would be able to read through the transcription terminators located downstream of the reporter genes and return to the cassette. If such readthrough occurred, the promoters located on the plasmid backbone would cause an increase in fluorescence in cells containing empty plasmids compared to plasmid-free cells, a phenomenon that we did not observe (Figure S1). In addition to the reporter cassette, the constructed reporter plasmids encode functions essential for plasmid maintenance, including a replication initiation mechanism, the *bla* gene, and the *hok-sok* system, all of which rely on active transcription. In addition, the plasmid backbones contain several promoters derived from their construction, although most of these remain unannotated. Some examples for comparison, the vector pPR9TT contains putative σ^70^-type promoters such as GTGATA N_17_ TAATGT (score 5.6) and TTAAG N_17_ TAAACG (score 4.63) with scores comparable to the test promoter used in this study (PnuoA, TTTACT N_17_ TAAAAT (score 4.3)). Importantly, the transcriptional terminators flanking the reporter cassette (Table S2, Figure 3) are strong enough to terminate any transcriptional read-through from these cryptic promoters, thereby avoiding spurious or artifactual induction of reporter gene expression.

Although the antibiotic resistance encoded from the plasmid persisted well in cells cultivated without antibiotics (Figure 2B), the other elements of the plasmids, including the expression of reporter genes, may cause the loss of the plasmid, resulting in a reduced copy number of reporter genes per cell. In this case, the cells grown without antibiotics as the selection marker should emit less light than the cells grown with antibiotics. Therefore, we cloned a version of the PnuoA promoter region, which contains a single promoter upstream of either the SYFP2 or Scarlet-I3 reporter gene in the BBR1-type and RK2-type reporter plasmids (Table S3, Figure 4A). The PnuoA promoter was selected as a medium-strong promoter active in both exponentially growing and stationary phase cells.^33^ Thereafter, the fluorescence of cells cultivated with and without penicillin G was compared (Figure 4). As the fluorescence profiles remained comparable between studied growth conditions (Figure 4B–E), we can conclude that the presence or absence of penicillin does not significantly alter fluorescence intensity within a minimum 18-h time frame, as is commonly applied in similar experimental setups.

**Figure 4:**
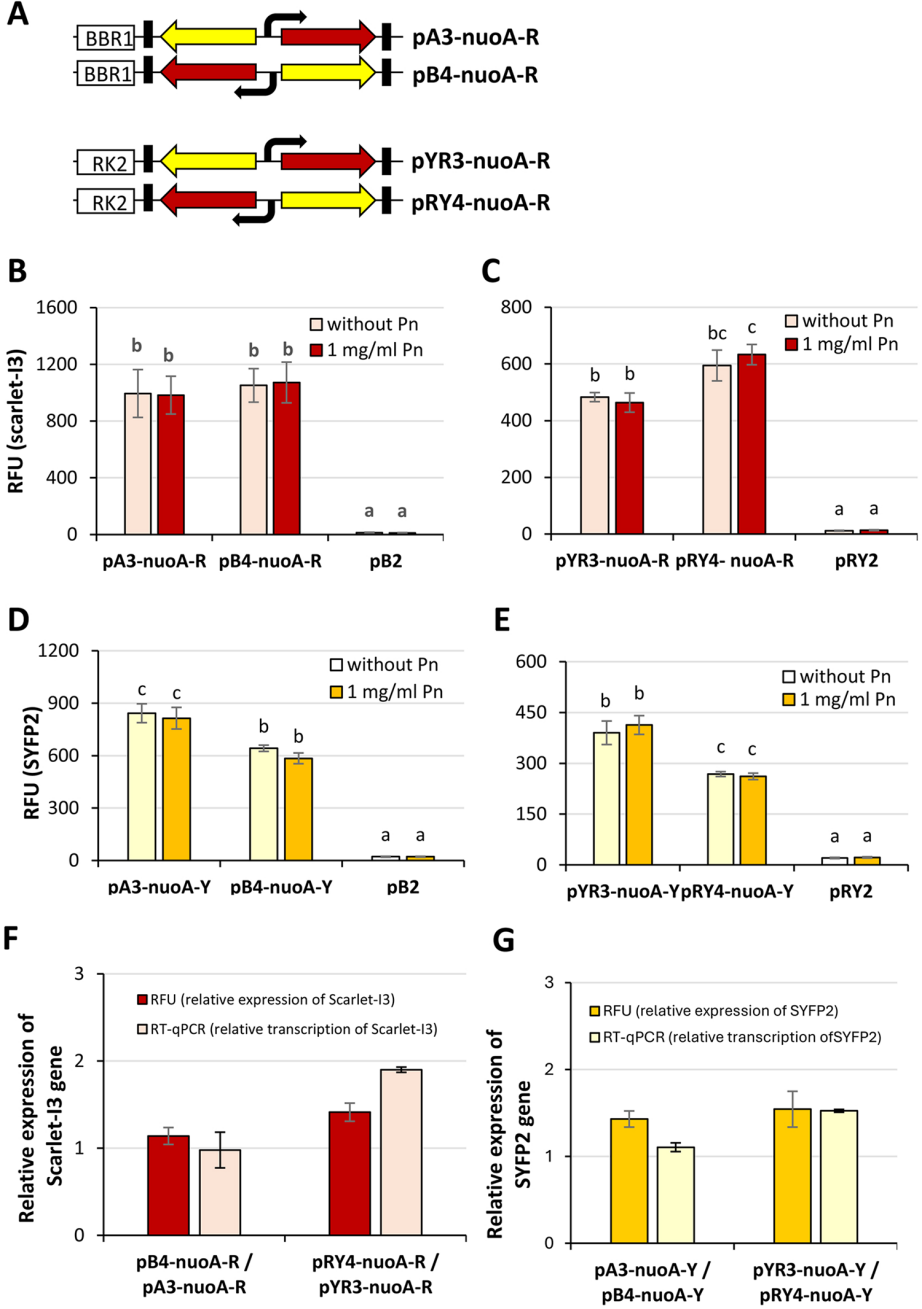
Fluorescence and mRNA analysis of *P. putida* PaW85 carrying PnuoA promoter-driven reporter plasmids using RT-qPCR. (A) Schematic representation of reporter cassettes, where the PnuoA promoter is oriented toward Scarlet-I3; the orientation toward SYFP2 is not depicted. The figure highlights *oriV* regions of BBR1 and RK2 (white boxes), transcriptional terminators flanking the reporter cassette (black boxes), the Scarlet-I3 gene (red arrow), and the SYFP2 gene (yellow arrow). A black arrow indicates the PnuoA promoter orientation. Plasmid names are displayed adjacent to their respective schematics. (B–E) Fluorescence measurements of *P. putida* PaW85 cells harboring the respective plasmids. Cultures were grown in LB medium for 18 h at 30 °C and 180 rpm, in the presence or absence of 1 mg/mL penicillin G (*n* = 9). (B, C) Relative fluorescence of Scarlet-I3. (D, E) Relative fluorescence of SYFP2. (F) As determined by RT-qPCR, Scarlet-I3 fluorescence and mRNA levels were normalized and (G) SYFP2 fluorescence and mRNA levels were normalized. Normalization was performed by comparing pA3 vs pB4 and pYR3 vs pRY4 plasmids (*n* = 3). Displayed values represent the arithmetic mean, with error bars indicating the 95% confidence interval. Multifactorial ANOVA was calculated from log10 values, with homogeneity groups indicated above the bars.

Subsequently, we were interested in whether the orientation of the reporter gene in the plasmid could affect reporter gene expression, since we had constructed a reporter system with bidirectional transcription. To achieve this, we inverted the reporter cassette in the BBR1 and RK2 plasmids, resulting in the pA3 and pB4 pairs, and the pYR3 and pRY4 pairs, respectively (Figure 3). Since the plasmid in front of the reporter genes is in exactly the same position, any possible difference in cell fluorescence can only be caused by the orientation of the reporter cassette. Indeed, the fluorescence intensity of the RK2-type plasmids was influenced by the orientation of the gene cassette. Specifically, cells harboring pRY4-nuoA-R exhibited stronger red fluorescence compared to those containing pYR3-nuoA-R (without penicillin, *p* = 0.454; with penicillin, *p* = 0.001), while yellow fluorescence was more pronounced in pYR3-nuoA-Y relative to pRY4-nuoA-Y (*p* < 0.001; Figure 4). Conversely, in BBR1-derived plasmids, red fluorescence intensity remained orientation-independent (*p* = 1), whereas yellow fluorescence was significantly higher in pA3-nuoA-Y than in pB4-nuoA-Y cells (without penicillin, *p* = 0.001, with penicillin, *p* < 0.001; Figure 4). These findings suggest that the orientation of the reporter cassette influences transcriptional output and fluorescent gene expression because the DNA sequence in front of the reporter gene remained the same, and the only difference was in the whole cassette’s orientation.

We investigated whether the fluorescence dependence on reporter cassette orientation could be attributed to differences in mRNA abundance. In other words, we assessed whether the orientation of the reporter cassette influenced reporter gene transcription. Indeed, when the cells were grown in the presence of 1 mg/mL of penicillin G, the differences in fluorescence observed in cells containing pYR3 and pRY4 (1.4-fold difference of Scarlet-I3 fluorescence and 1.5-fold difference of SYFP2 fluorescence) matched surprisingly well with the difference in mRNA levels (1.9 for Scarlet-I3 and 1.5 for SYFP2; Figure 4). Similarly, in cells containing pA3 and pB4 plasmids, the emitted fluorescence was correlated with the corresponding reporter gene’s mRNA abundance. Specifically, for Scarlet-I3 in BBR1 plasmids, neither fluorescence (1.1-fold) nor mRNA levels (1.0-fold) were affected by the orientation of the reporter cassette. However, unlike its mRNA levels (1.1-fold difference), SYFP2’s fluorescence (1.4-fold difference) was significantly higher in cells carrying pA3-nuoA-Y than those with pB4-nuoA-Y (Figure 4).

In conclusion, we have constructed both low-copy-number and medium-copy-number plasmids that are maintained in *P. putida* and do not affect the results when cells are grown for a short time without plasmid-selecting antibiotics.

### Protein Half-Life Reducing Tags for More Accurate Gene Expression Regulation Assessment

To better assess gene expression, it is necessary to shorten the half-life of reporter proteins to reflect the transcriptional events occurring in the cell more accurately. Thus, we have constructed reporter plasmids carrying genes with a tag sequence ensuring the shorter half-life of FPs to facilitate more precise assessment of gene expression. The protein tags with different DNA sequences, but with a similar reduction effect on reporter protein half-life, were inserted into the same reporter cassette. We used AAV/ASV2 and LVA/LVA2 (LVA and LVA2 encoding the same peptide) tags to shorten the half-life of reporter proteins. Previous experiments have shown that these tags reduced the half-life of GFP in *E. coli* to 60 and 40 min, respectively.^34^ While the half-life of each reporter protein can vary depending on the context, our goal was to shorten the half-life of Scarlet-I3 and SYFP2 to minimize protein accumulation and allow for more precise measurement of gene expression levels. For this purpose, we used the PnuoA promoter with plasmids pA3 and pB4 (without tags), pA7 and pB8 (with AAV/ASV2 tags), and pA11 and pB12 (with LVA/LVA2 tags, Figure 3, Table S3). We chose medium-copy-number plasmids for the experiment because a strong signal is needed to assess the suitability of the tags. With a weak signal, changes in fluorescence may not be visible.

It was found that, while the tag-free variants exhibited visible protein accumulation (Figure 5), the AAV/ASV2-tagged reporters reflected gene expression more accurately, and changes in gene expression during the stationary phase were more clearly observable.

**Figure 5:**
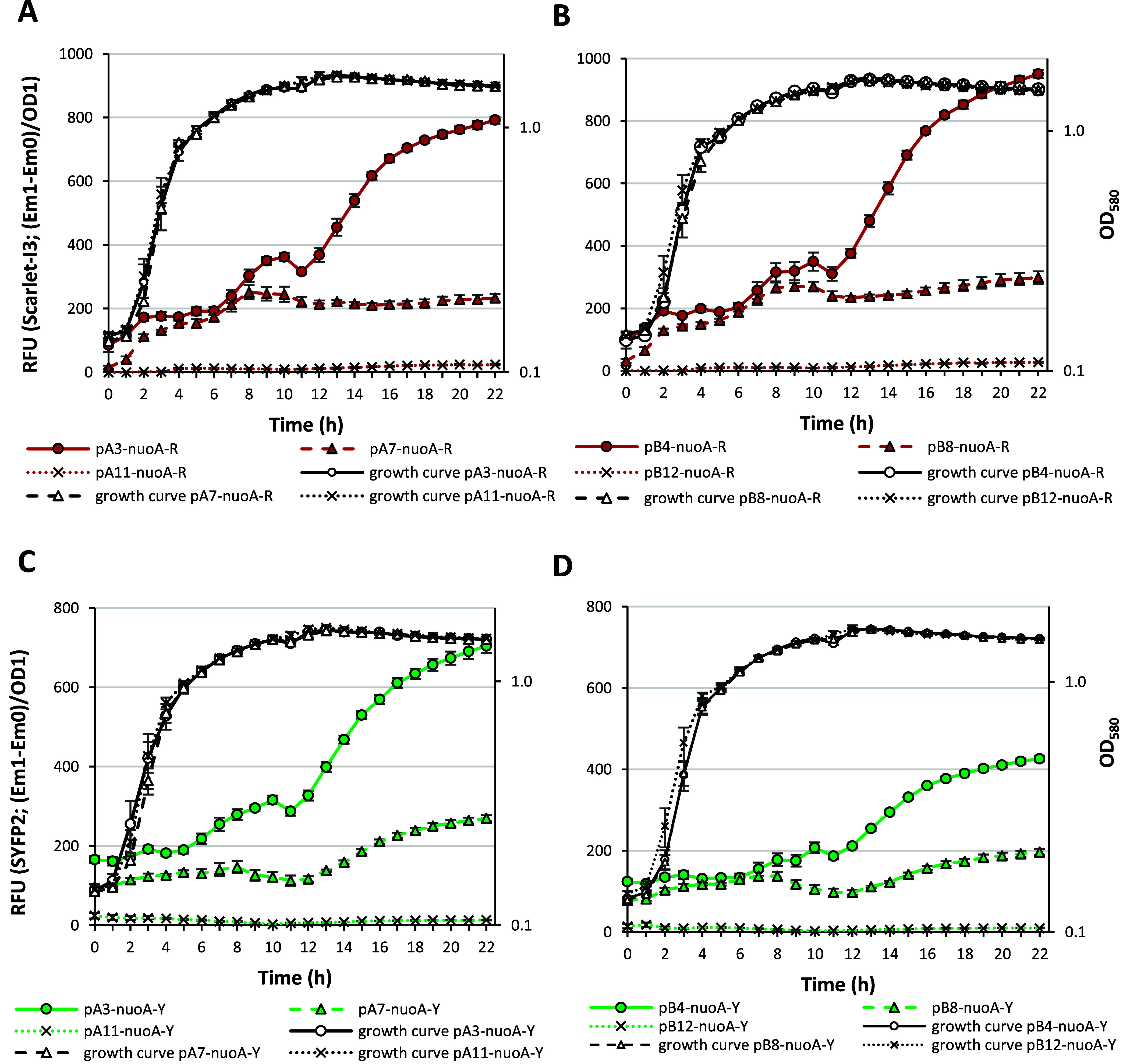
Fluorescence of reporter proteins with tags in *P. putida* PaW85. Cells were grown in LB supplemented with 1 mg/mL penicillin G for 22 h in a microtiter plate, and absorbance at 580 nm and fluorescence intensity at the appropriate wavelengths were measured every 20 min (growth, shown as black lines). In the top panel (A, B), the relative fluorescence of Scarlet-I3, and in the bottom panel (C, D), the relative fluorescence of SYFP2, is shown, measured every 1 h. In the left panel (A, C), the development and fluorescence of cells carrying pA plasmids, and in the right panel (B, D), cells carrying pB plasmids. Colored lines represent fluorescence. The *P. putida* PaW85 was transformed with constructs of tag-free reporter genes (pA3 and pB4), with constructs carrying reporter genes Scarlet-I3-AAV and SYFP2-ASV (pA7 and pB8), and Scarlet-I3-LVA and SYFP2-LVA2 (pA11 and pB12). The orientation of the PnuoA promoter is written by nuoA-R (PnuoA is in front of the Scarlet-I3 gene variants) and nuoA-Y (PnuoA is in front of the SYFP2 gene variants). Displayed values represent the arithmetic mean, with error bars indicating the 95% confidence interval.

In contrast, the LVA tag caused such rapid degradation of the reporter protein that gene expression could not be assessed, as the fluorescence signal became similar to the background fluorescence (Figure 5). However, the pA constructs (compare the colored lines in Figure 5A,C) exhibited similar fluorescence for both reporter proteins compared to plasmids in the pB constructs (compare the colored lines in Figure 5B,D), which were more sensitive to the orientation of the reporter cassette. Thus, the pA plasmids provided a more convenient option for analysis.

### Assessment of Gene Expression Activation and Repression Using Reporter Plasmids

To assess the potential for transcriptional activation, we used the PnuoA promoter region, as it is known that Fis increases transcription from this promoter.^33^ Additionally, we used the *P. putida* strain F15,^35^ which contains a chromosomal *fis* gene under the control of the IPTG-inducible Ptac promoter. In other words, adding IPTG to the culture medium increases the concentration of Fis in the cells, and transcription from the PnuoA promoter should be enhanced. Both BBR1-origin plasmids (pA3 and pB4) and RK2-origin plasmids (pYR3 and pRY4) showed increased fluorescence in cells in the presence of IPTG (Figure S2), indicating increased level of transcription of the reporter genes. Thus, all tested plasmids were suitable for the detection of transcriptional activation.

To evaluate the potential for detecting the transcriptional repression, we selected the promoter region of the *lapF* gene, which contains a single promoter that is RpoS-dependent and whose transcription is repressed by Fis.^36^ This promoter region was cloned between the reporter genes, and we focused our analysis on plasmids pA3-lapF-R/Y, pA7-lapF-R/Y, pYR3-lapF-R/Y, and pYR7-lapF-R/Y (Figures 6 and S3). We included AAV/ASV2-tagged reporters in this experiment because we wanted to evaluate the utility of reporter plasmids in cases of particularly weak repression signals (especially weak promoters). Therefore, we paid special attention to the pYR7 constructs.

**Figure 6:**
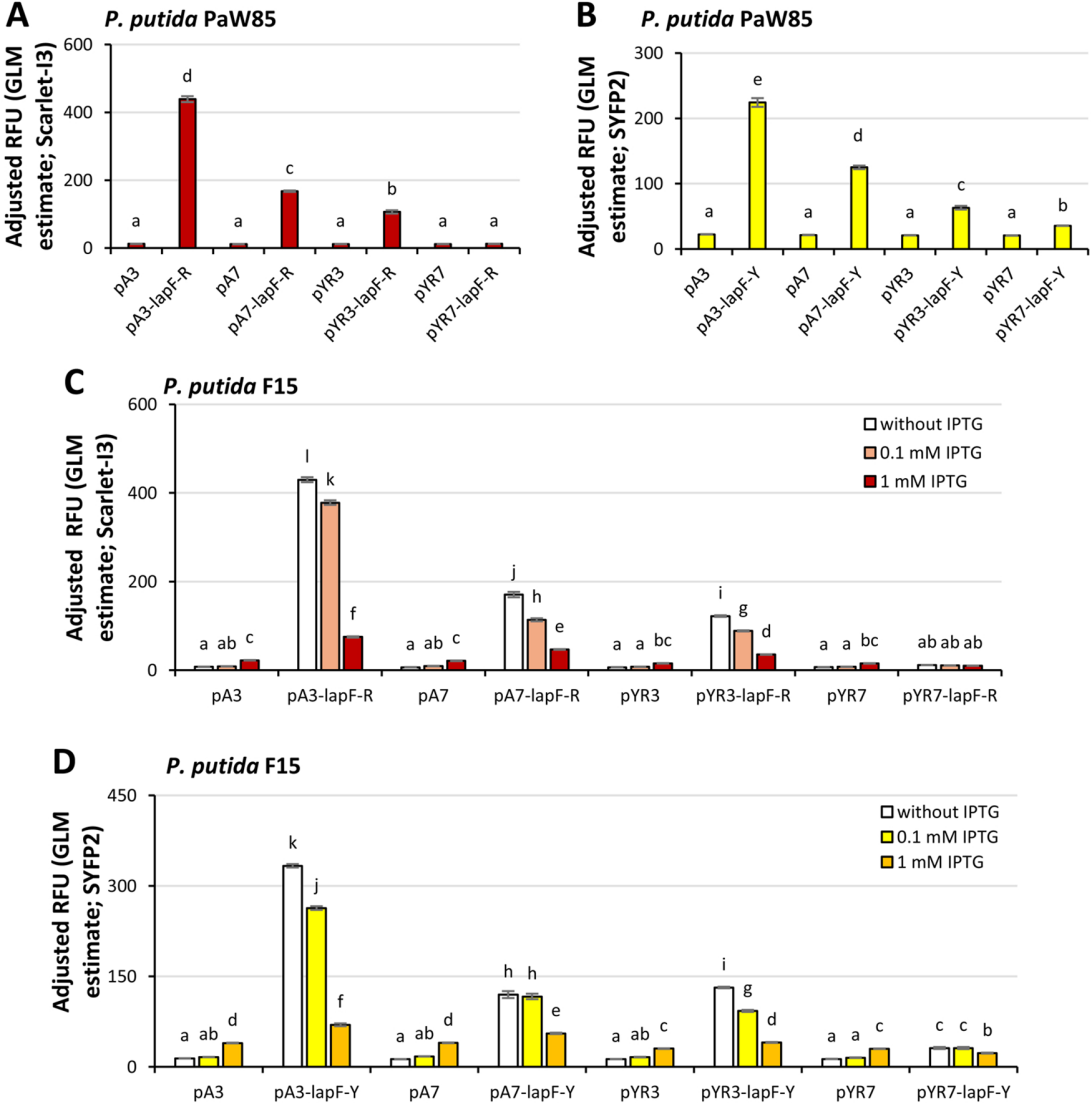
Evaluation of transcriptional repression using the developed reporter systems in *P. putida*. *P. putida* PaW85 (A, B) and strain F15 (C, D) carrying the respective plasmids were cultivated for 22 h in LB medium supplemented with 1000 μg/mL penicillin G in a microtiter plate. Optical density at 580 nm and fluorescence of Scarlet-I3 and SYFP2 were measured every 20 min. For *P. putida* F15, IPTG was added at final concentrations of 0.1 mM, 1 mM, or grown without IPTG supplementation. The data from 15 to 22 h were analyzed using the General Linear Model (GLM), and the adjusted relative fluorescence values are presented. Homogeneity groups are indicated above the bars.

To assess the feasibility of detecting transcriptional repression, we employed two approaches: (i) we compared the fluorescence of cells carrying pYR (RK2, low-copy-number plasmid) and pA (BBR1, medium-copy-number plasmid) constructs. This experiment focused on determining the earliest detectable fluorescence signal in cells. (ii) We evaluated the transcriptional inhibition by inducing the repressor’s expression in *P. putida* strain F15.

Surprisingly, fluorescence detection in wild-type *P. putida* PaW85 occurred at a similar growth phase for both pA3 and pYR3, specifically, when the growth speed was decreasing or in the stationary phase (8–9 h of measurement; Figure S3). This suggests that, despite the higher copy number of BBR1 plasmids, the release of gene transcription from Fis repression and transcriptional activation by RpoS occurs at a similar growth phase as with RK2 plasmids (Figure S3). However, the maximal fluorescence intensity in BBR1 plasmid-carrying cells was higher than in RK2 plasmid variants, which improves the ability to detect changes in gene expression (Figures 6 and S3). Notably, when the AAV/ASV2 tag was used, the fluorescence of RK2 plasmid-containing cells was either undetectable or too close to noise, making the assessment of expression changes challenging or close to impossible (see pYR7-lapF-R and pYR7-lapF-Y in Figure 6A,B).

In *P. putida* strain F15, the repression was detected in all constructs except pYR7-lapF-R (Figure 6C) and pYR7-lapF-Y, which emitted low fluorescence that was close to the background fluorescence of bacteria (Figure 6D). In the case of pA7-lapF-Y (BBR1 plasmid), a higher IPTG concentration (1 mM) was required to detect fluorescence reduction (Figure 6D). However, repression would probably be observable at intermediate IPTG concentrations between 0.1 and 1 mM. Thus, our results indicate that the other medium-copy-number plasmids can be used to study transcriptional repression. However, using tags to shorten reporter protein half-life may hinder or prevent weak signal detection in low-copy-number (RK2) plasmids (Figure 6).

To assess the simultaneous expression of two fluorescent reporters within the same cell, we fused promoter regions PnuoA and PlapF to create a promoter area with divergently oriented promoters and cloned it into pA3 and pA7 (Figure 7A, Table S3). The fluorescence profiles of cells expressing individual promoters versus the synthetic fusion promoter were comparable. PnuoA functioned as a strong, semiconstitutive promoter active across all growth phases; in contrast, PlapF was predominantly active during the growth deceleration and stationary phases (Figures 5, 7 and S3). Notably, the fluorescence signal in pA7-containing cells increased more gradually than in pA3-containing cells, highlighting the AAV/ASV tag’s decreased fluorescence effect due to reduced reporter protein stability (Figure 7). These findings demonstrate that the developed reporter system enables simultaneous monitoring of transcription from two different promoters.

**Figure 7:**
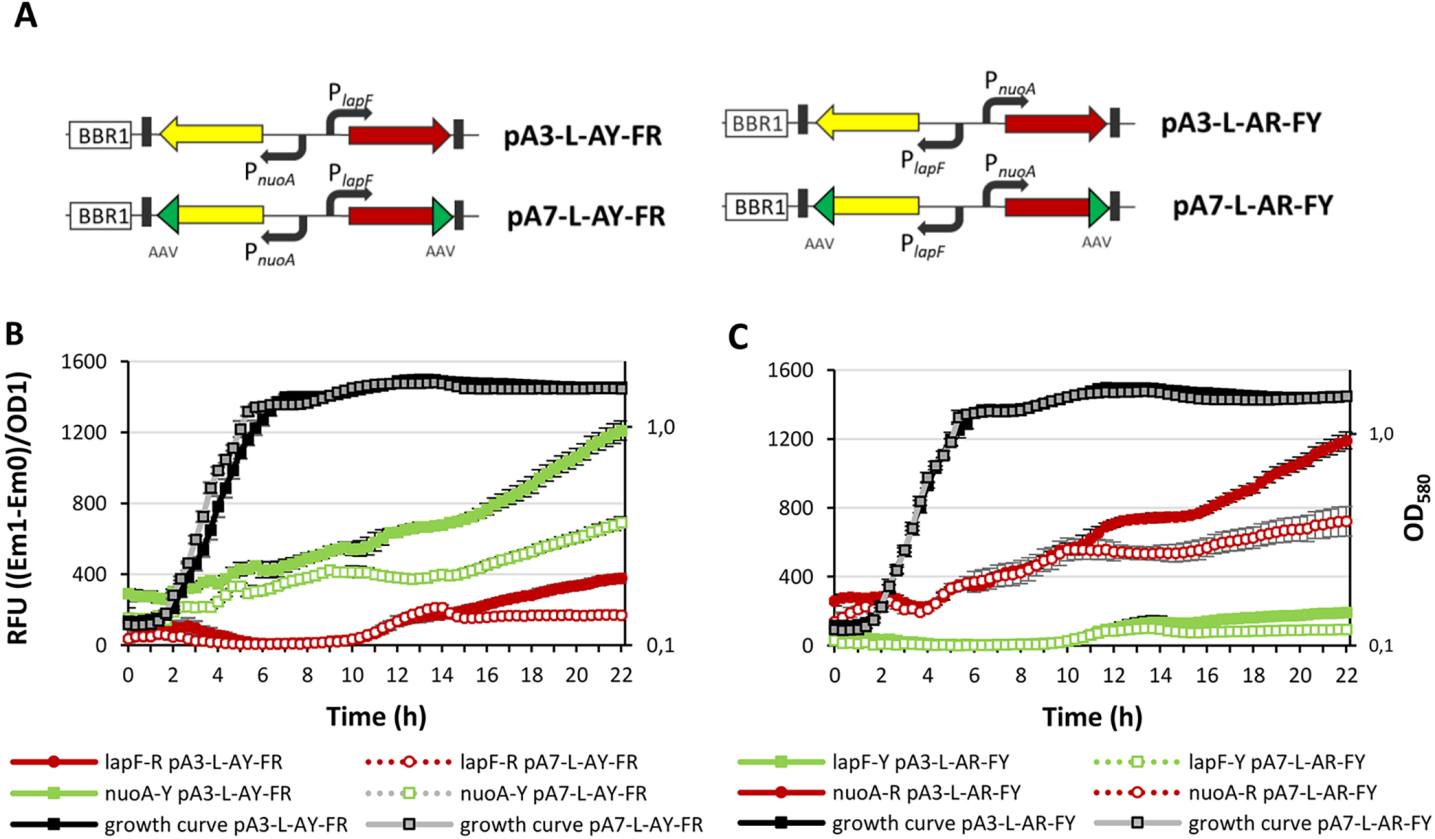
Fluorescence of *P. putida* PaW85 cells carrying plasmids with an artificially constructed PnuoA-PlapF divergently oriented promoters. Cells were grown for 22 h in LB medium supplemented with 1000 μg/mL penicillin G in a microtiter plate, with optical density at 580 nm and fluorescence measured every 20 min. (A) Schematic representation of the constructs carrying the divergently oriented PnuoA-PlapF promoters (promoters indicated by black arrows), cloned between reporter genes Scarlet-I3 (red arrow) and SYFP2 (yellow arrow). The plasmids used (pA3 and pA7) contain the BBR1 origin (white box), with pA7 additionally carrying an AAV peptide tag (green arrows) at the 3′ end of the reporter genes to reduce protein half-life. Plasmid names are displayed to the right. (B) Fluorescence of pA3 and pA7 plasmid-containing cells, where PlapF drives Scarlet-I3 expression. Red lines represent Scarlet-I3 fluorescence (PlapF-driven), while green lines represent SYFP2 fluorescence (PnuoA-driven). Solid lines indicate the fluorescence of pA3-L-AY-FR, and dashed lines indicate the fluorescence of pA7-L-AY-FR. Black and gray lines show growth curves for pA3-L-AY-FR and pA7-L-AY-FR cells, respectively. (C) Fluorescence of pA3 and pA7 plasmid-containing cells, where PlapF drives SYFP2 expression. Red lines represent Scarlet-I3 fluorescence (PnuoA-driven), while green lines represent SYFP2 fluorescence (PlapF-driven). Solid lines indicate the fluorescence of pA3-L-AR-FY, and dashed lines indicate the fluorescence of pA7-L-AR-FY. Black and gray lines show growth curves for pA3-L-AR-FY and pA7-L-AR-FY cells, respectively. Displayed values represent arithmetic means, with error bars indicating the 95% confidence interval. Statistical analysis is not performed.

### Application of the Reporter System for Studying Gene Expression in Sessile (Biofilm-Associated) *P. putida*

Our next objective was to assess the usability of the reporter system for visualization of gene expression dynamics in biofilm-associated *P. putida*. Namely, we were interested in whether the *lapF* expression could differ in biofilm layers, as this gene is known to be expressed in planktonic cells predominantly during the stationary phase,^36,37^ which should be characteristic of the deeper layers of the biofilm. For that purpose, the pBVS2–7-AY-FR (encoding Scarlet-I and mVenus pair, Table S3) was used instead of pA7-AY-FR, since the signal of Scarlet-I3 encoded from pA7-AY-FR was difficult to detect by confocal microscopy (Figure 8).

**Figure 8:**
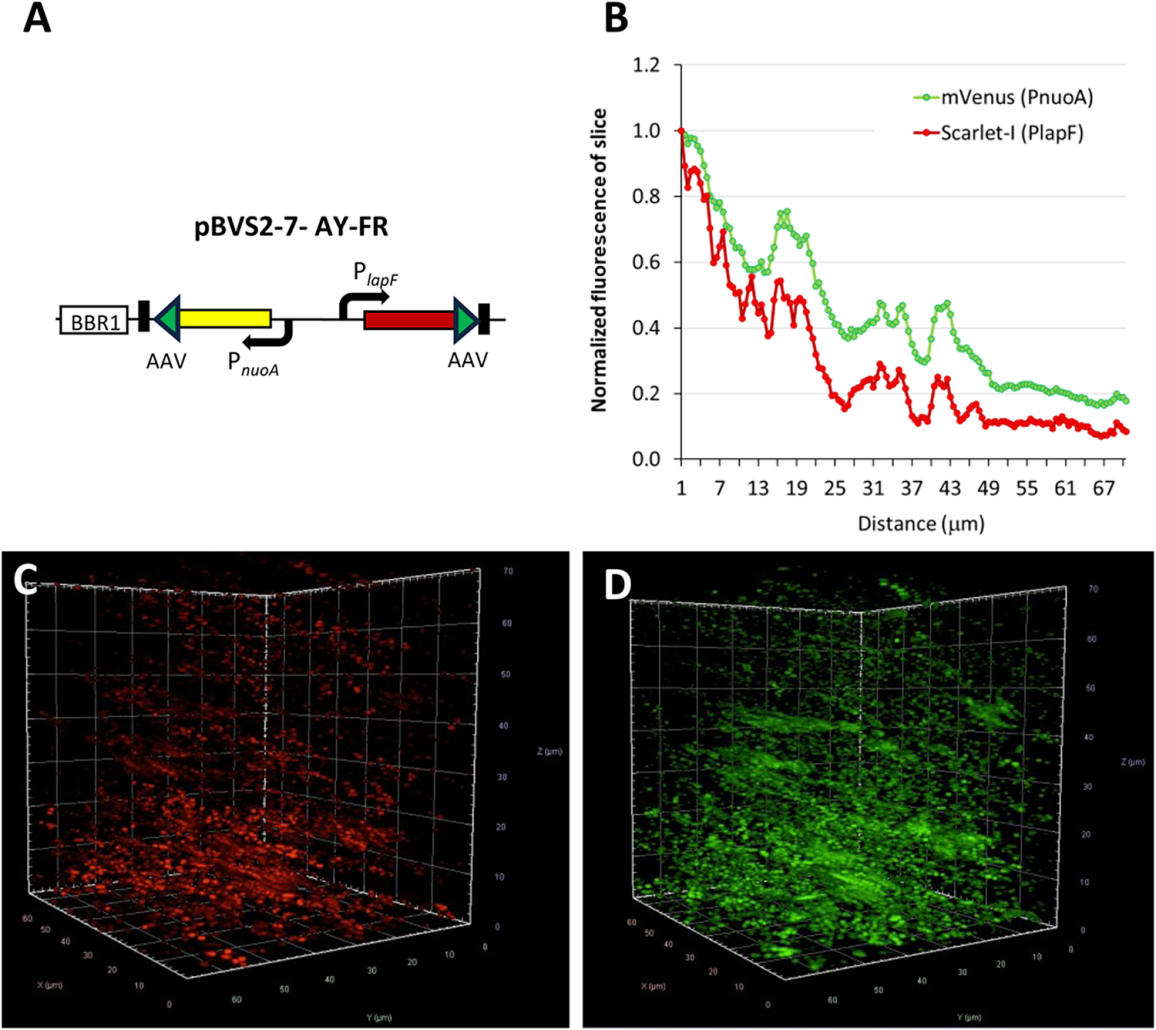
Visualization of gene expression in *P. putida* PaW85 [pBVS2–7-AY-FR] biofilm. (A) Schematic representation of the reporter cassette used in the plasmid. The BBR1 *oriV* is indicated by a white box, the mVenus-AAV gene by a yellow arrow, and the Scarlet-I-AAV gene by a red arrow. The green triangle represents the AAV sequence. The direction of promoters is shown with black arrows, while transcription terminators are depicted as black boxes. (B) Normalized fluorescence intensity was calculated for each slice in the z-stack (0.5 μm spacing). Scarlet-I-AAV (red line; excitation: 561 nm: 5%, emission: 600 nm) and mVenus-AAV (green line; excitation: 514 nm: 2%, emission: 540 nm) were normalized separately. (C) *P. putida* PaW85 [pBVS2–7-AY-FR] biofilm with Scarlet-I-AAV (PlapF) visualization. Axes represent distance in micrometers. (D) *P. putida* PaW85 [pBVS2–7-AY-FR] biofilm with mVenus-ASV (PnuoA) visualization. Axes represent distance in micrometers.

A 24-h-old *P. putida* biofilm formed in LB medium without penicillin G was examined using fluorescence microscopy (Axio Observer.Z1/7). To visualize the cells within the biofilm, fluorescence signals were captured from a 70 μm-thick layer (Figure 8C,D), summing fluorescence intensity from 0.5 μm-thick layers separately over a 67.34 × 67.34 μm. The fluorescence intensities of Scarlet-I-AAV and mVenus-AAV were quantified separately and subsequently normalized. To compare fluorescence across biofilm layers, the summed fluorescence intensity of each layer was divided by the highest observed summed fluorescence value (Figure 8B). As shown in Figure 8B,C, the fluorescence intensity of Scarlet-I-AAV (driven by PlapF) sharply decreases at approximately 20 μm. In contrast, the fluorescence intensity of mVenus-AAV (driven by PnuoA) declines more slowly in general (Figure 8B,D). Thus, these results indicate that the mVenus-Scarlet-I reporter system is suitable for investigating gene expression dynamics across different biofilm layers.

### Construction of Excludon-Based Test Plasmid pC13A-AAV

Excludon, competition of RNA polymerases for promoters, the collision of RNA–RNA or RNA-DNA polymerases, etc., can regulate the expression of genes that are located close together.^14,38^ We were interested in whether it is possible to mimic such regulatory possibilities with the created reporter system by synthesizing the sense and antisense strands of a gene in *P. putida*.

To assess the feasibility of an excludon-based regulatory system, we designed a test plasmid pC13A-AAV in which a reporter gene was flanked by inducible promoters that could drive the expression of both the sense (mRNA) and antisense (asRNA) transcripts (Table S3, Figure 9A). The RK2-based plasmid facilitated robust transcription of the Scarlet-I3-AAV reporter gene in response to effector molecules while avoiding excessive metabolic burden on the host cells. The Scarlet-I3-AAV reporter gene was placed under the control of two inducible promoters: (i) Ptac promoter, inducible by IPTG, inducing sense strand (mRNA), (ii) *Pm* promoter, inducible by m-toluate, inducing antisense strand (asRNA; Table S3, scheme in Figure 9). Transcription terminators flanked the reporter gene and promoters, while regulator genes (*lacI* and *xylS*) were placed outside the expression cassette, followed by transcriptional terminator sequences (Table S3, scheme in Figure 9).

**Figure 9:**
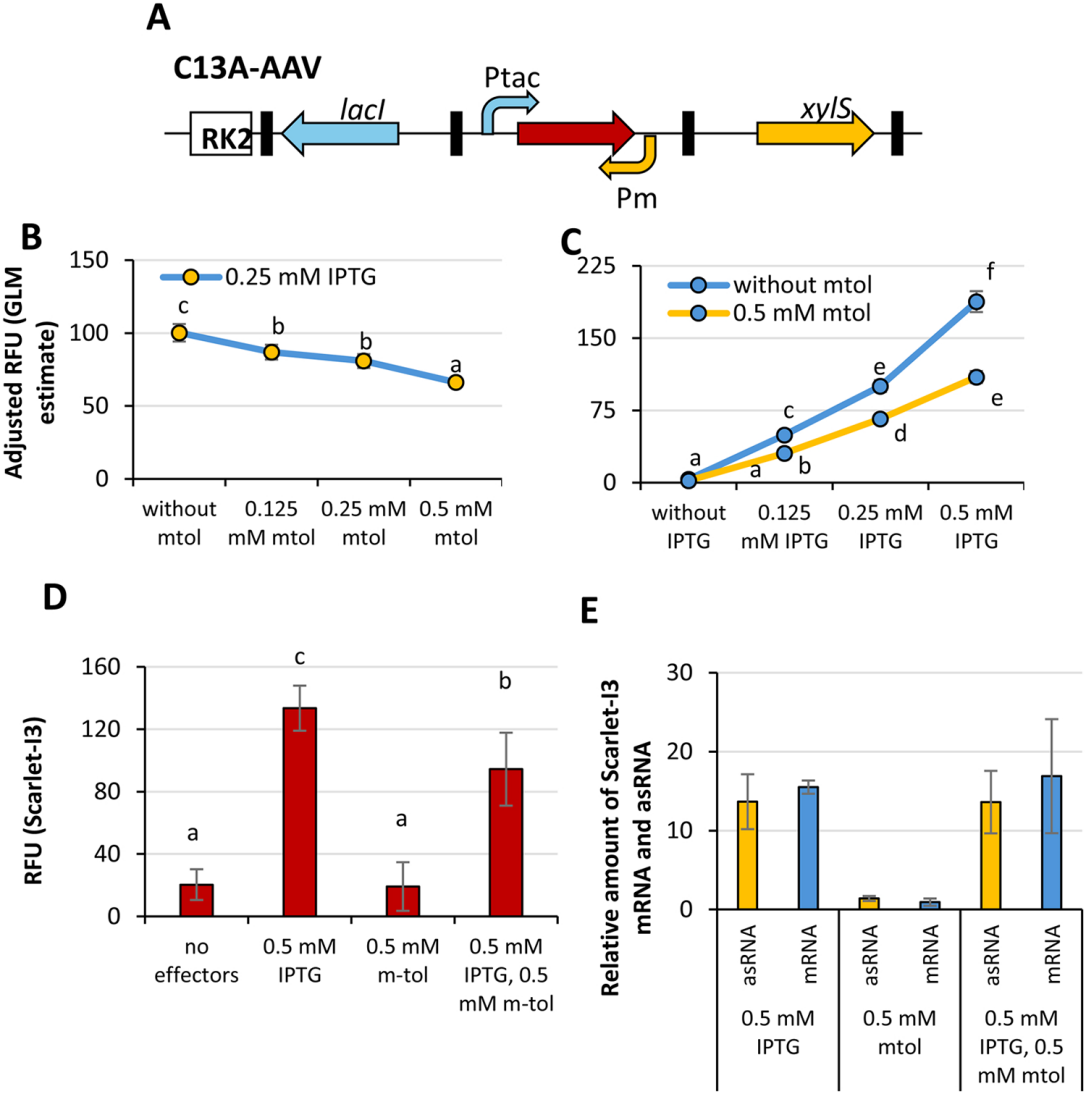
Assessment of excludon possibility using test plasmid pC13A-AAV in *P. putida* PaW85. (A) The scheme of the reporter system, where the replication origin, genes, and promoters are indicated. Black boxes indicate transcriptional terminators. (B, C) Cells were cultivated in LB + 1000 μg/mL penicillin G in a 96-well microtiter plate. Optical density at 580 nm (OD580) and fluorescence (570 (±20), 600 (±20)) were measured every 20 min. (B) The data in the upper panels of each block were analyzed using the General Linear Model (GLM), and the adjusted relative fluorescence values are presented in the figure. The adjusted relative fluorescence is shown for cultures supplemented with 0.25 mM of the effector IPTG that induces reporter gene mRNA synthesis, along with varying concentrations of the m-toluate that induces asRNA synthesis. (C) The adjusted relative fluorescence (GLM) for different combinations of effector concentrations. (D, E) Cells were grown in 5 mL LB medium supplemented with 1 mg/mL penicillin. Expression of Scarlet-I3 mRNA was induced by the addition of 0.5 mM IPTG, and asRNA expression was induced by 0.5 mM m-toluate. Following induction, cells were incubated for an additional 6 h prior to (D) end point fluorescence measurements and (E) RNA extraction (*n* = 3). Fold change of Scarlet-I3 mRNA and asRNA levels relative to the untreated sample. The Pfaffl method was used to calculate the change in transcript levels from by RT-qPCR data. The error bars indicate the 95% confidence interval. Homogeneity groups obtained by the posthoc Bonferroni test are also presented.

### Experimental Validation of Excludon Reporter System Functionality

To validate the functionality of the excludon reporter system, we first evaluated the basal gene expression from the inducible promoters by comparing the fluorescence of *P. putida* PaW85 carrying pC13A-AAV in LB medium without adding effectors (Figure S4A,B). As a negative control, we used the plasmid pYR7, which is also an RK2-type plasmid but lacks promoters upstream of the reporter genes (Figure S4A,B). The cells emitted slightly more red fluorescence than the negative control pYR7 (Figure S4A). Nevertheless, when analyzing relative fluorescence across different growth phases (Figure 9B), the extent of promoter leakage appeared minimal and did not significantly affect the investigation of excludon function. This is because the emergence of antisense transcripts should, in the case of the excludon mechanism, decrease cellular fluorescence regardless of basal promoter activity.

To induce the synthesis of Scarlet-I3 mRNA and asRNA, we supplemented the growth medium with different concentrations of IPTG and m-toluate (Figures 9 and S4C). We expected fluorescence intensity to decrease to background levels upon sufficient induction of the asRNA, indicating effective translational repression.

Reporter gene Scarlet-I3-AAV in this context acts as a gene of interest whose expression we are trying to induce using the Ptac or reduce by activating transcription from *Pm*, which promotes antisense synthesis. Fluorescence intensity decreased when m-toluate was added to the medium, as the *Pm* promoter was positioned downstream of the reporter gene to initiate the synthesis of the antisense strand. Furthermore, differences in fluorescence intensities were observed upon mRNA synthesis induction; whenever m-toluate was added to the medium in addition to IPTG, the cells fluoresced less than cells grown without m-toluate (Figures 9B,C and S4C). This clearly showed that transcription initiation from the *Pm* promoter prevents Scarlet-I3 expression. In addition, the fluorescence of the cells started to decrease in the stationary phase, indicating that the reporter system can also measure the decrease in expression (Figure S4C).

To assess the relative abundance of RNA upon induction, we performed RT-qPCR analysis. Cells were grown in 5 mL of LB medium to the exponential phase, after which effectors were added; the negative control received no effector. Cultures were incubated for an additional 6 h prior to RNA extraction. We shortened the incubation time, compared to the time required to reach maximal fluorescence signal, as RNA is produced earlier than the conformationally mature reporter protein and is likely subject to more rapid degradation.

Scarlet-I3 mRNA transcription was induced with 0.5 mM IPTG, while synthesis of the antisense RNA (asRNA) was induced with 0.5 mM m-toluate. If Scarlet-I3 expression were controlled via an excludon mechanism, the mRNA levels would be expected to remain comparable between IPTG-only and IPTG + m-toluate conditions. The observed reduction in fluorescence likely reflects the formation of mRNA–asRNA duplexes, which interfere with translation. Indeed, the relative amounts of Scarlet-I3 mRNA were similar under both IPTG-only and IPTG + m-toluate conditions (Figure 9D,E).

Unexpectedly, we did not detect asRNA in cells grown with m-toluate as the sole effector, whereas asRNA was readily detectable in cells cultivated with IPTG alone, despite the absence of an asRNA effector, thereby indicating leakiness of the *Pm* promoter (Figure 9E). Additionally, the relative abundance of asRNA was comparable between the IPTG-only and IPTG + m-toluate conditions, although the fluorescence of cells decreased (Figures 9D and S4C). A likely explanation is that asRNA was transcribed in m-toluate-only cultures but rapidly degraded due to its noncoding nature and lack of ribosome-mediated protection. In contrast, in the presence of mRNA, asRNA synthesized from a leaky *Pm* promoter may form protective duplexes with the mRNA of the Scarlet-I3 gene.

Nevertheless, duplex formation appears to be limited. The amount of asRNA in the IPTG + m-toluate condition was not higher than in the IPTG-only condition, suggesting that duplex formation is dynamic and not saturable. Consistent with this, fluorescence levels decreased upon addition of m-toluate to IPTG-induced cultures (Figure S4C), while asRNA levels remained unchanged (compare the 0.5 mM IPTG and 0.5 mM IPTG + 0.5 mM m-toluate conditions in Figures 9D,E).

In summary, it is possible to repress gene expression from a reporter plasmid pC13A-AAV with an inverse promoter *Pm*, and this is likely to occur through the formation of an mRNA and asRNA duplex, excludon.

## Discussion

Constructing and validating a fluorescence-based reporter system adapted for *P. putida* would be valuable for studying transcriptional regulation in this metabolically complex bacterium. Our system overcomes several important limitations that have hindered the use of traditional reporter systems in *P. putida*, such as high autofluorescence of these bacteria, fluorescence of complex growth medium components that overlap with the signal from the reporter system, and invasive measurement methods that necessitate cell disruption.

### Selection of Fluorescent Proteins for Bidirectional Promoter Areas

The intrinsic fluorescence caused by the autofluorescence of *P. putida* and the components of LB medium, particularly in the green spectral range, has posed a significant challenge in designing a suitable reporter system. As a result, we excluded fluorescent proteins with excitation or emission maxima below 530 nm from further consideration (Figure 1), due to signal interference from the background autofluorescence.

Two suitable monomeric FP pairs were selected: mVenus and Scarlet-I, with reported maturation times of 17.6 and 36 min, respectively, and SYFP2 and Scarlet-I3, with maturation times of 4.1 and 2.0 min, respectively (Table S1, Figure 1).^26^ Naturally, FPs with shorter maturation times are preferred for studying gene expression dynamics, as they allow more accurate temporal resolution of transcriptional changes. For this reason, reporter vectors based on SYFP2 and Scarlet-I3 (pA, pB, pYR, and pRY) are particularly suitable for promoter probe plasmids (Table S3, Figure 3). However, due to the short photostability of Scarlet-I3, the signal is often weak or unstable in microscopy applications.^27^ Therefore, the mVenus and Scarlet-I pair, implemented in the pB_VS2 vector series (Table S1, Figure 8), is preferred for fluorescence imaging.

### Minimizing Basal Fluorescence, Orientation-Dependent Effects and Asymmetric Expression

The reporter system must exert minimal interference on the target’s expression. Therefore, we considered several factors: (i) unintended transcriptional read-through from the adjacent reporter gene or plasmid backbone elements, (ii) orientation-dependent effects of the reporter cassette, and (iii) minimizing the possibility that insertion of the promoter region under study would introduce asymmetrical gene expression from reporter genes.

We prioritized minimizing basal fluorescence originating from the reporter vector by designing reporter plasmids where transcription is initiated exclusively from the target promoter. The SYFP2–Scarlet–I3 reporter gene pair exhibited exceptionally low background fluorescence, yielding levels comparable to those observed in plasmid-free cells (Figures 1 and S1). Consequently, vectors based on this fluorescent protein pair (pA and pYR) enable the detection of even weak transcriptional signals.

Accurate detection of low expression levels is particularly challenging when studying weak promoters. To address this, we deliberately used both a low-copy-number origin (RK2) and a medium-copy-number origin (BBR1), allowing flexibility in vector performance. Surprisingly, we observed that the cellular burden imposed by plasmids was influenced more by the assembly context of the construct than by the selection marker or the origin of replication (Figure 2). Furthermore, BBR1-based plasmids demonstrated excellent persistence in cells, even without antibiotic selection, during short-term assays (Figure 4). As gene expression monitoring experiments typically span 16–24 h in the laboratory, this feature enables the use of BBR1-based reporters in scenarios when antibiotics cannot be added to the growth medium. In contrast, RK2-based plasmids may require stabilization via a toxin-antitoxin module for plasmid maintenance without selection (Figure 2B).

The most unexpected result was that the orientation of the reporter cassette influenced reporter gene expression within the plasmid (Figure 4). While one possible explanation is the presence of transcriptional read-through or unintended initiation from other plasmidial promoters that are located outside the reporter cassette, which could enhance gene expression in one direction, our data did not support this hypothesis. Specifically, such orientation-dependent differences in expression should persist even in the “empty” vector lacking an inserted promoter between the reporter genes; however, this was not observed in our control experiments (Figure S1). Moreover, since the entire reporter cassette (∼1.7 kb) was inverted within the plasmids maintaining the same promoter region and DNA of opposite gene in front of reporter gene (identical ∼ 1 kb upstream DNA region, Figure 3) this excludes the possibility that the observed transcriptional differences could be a consequence of classical transcriptional regulation mechanisms, such as transcriptional regulators or transcription readthrough from external promoters (Figure S1). Instead, our findings suggest that expression of the studied reporter genes could be influenced by collisions between RNA polymerase (RNAP) and DNA polymerase (DNAP) during replication and transcription. It is relevant especially in the case of RK2 plasmids, where RK2 replication is unidirectional.^39,40^ In the case of plasmid pYR, the direction of plasmid replication coincides with the transcriptional orientation of the SYFP2 gene. In contrast, pRY plasmids align with the transcription of the Scarlet-I3 gene, increasing expression of Scarlet-I3 (Figure 4). In the case of BBR1 plasmids, there is no clear evidence about uni- or bidirectional replication, and the dependence of gene expression on the direction of replication fork movement in the case of pA and pB plasmids was not as pronounced as observed for RK2-based plasmids (Figure 4). One possible explanation is that the higher copy number of BBR1 may mask any transcriptional asymmetry arising from collisions between DNA and RNA polymerases.

For subsequent experiments, we selected the pA (BBR1) and pYR (RK2) series reporter plasmids, as pA showed less orientation-dependent variation in reporter gene expression compared to the pB series (Figure 4). The pYR plasmid was chosen because its orientation was identical to pA. However, considering that reporter gene expression may depend on the orientation of the reporter cassette, the pB (BBR1) and pRY (RK2) plasmids can also be used, provided that orientation effects are taken into account during experimental design.

To minimize asymmetric gene expression, we reduced the length of upstream noncoding DNA preceding the reporter genes, thereby limiting potential impacts on mRNA secondary structure that could interfere with translation efficiency. To this end, a *Bam*HI recognition site was introduced 10 bp upstream of each reporter gene’s ribosome binding site (RBS) to facilitate insertion of only the promoter region into the reporter plasmid (Tables S2 and S3). In plasmid variants designed for translational fusions between the gene of interest and the reporter gene, the ATG start codons of the divergent reporter genes were replaced with the *Bam*HI sequence (GGATCC), allowing for in-frame fusion of the gene of interest at the desired position (Table S3).

### Interpretation of High Relative Fluorescence in Early Growth Phase

The interpretation of results was complicated by elevated relative fluorescence units (RFU) observed during the lag and early to-mid exponential phases. These may be two possible reasons: (i) a mathematical artifact or (ii) residual fluorescence derived from the stationary phase culture. Inoculation with overnight-grown cultures results in low cell density (OD_580_ = 0.1–0.3), and dividing fluorescence by such small OD values can lead to an apparent 10-fold increase. This effect is particularly problematic when weak signals are evaluated. Figure S4B describes this situation, where a low fluorescence signal divided by very low OD values leads to high relative fluorescence.

To address this issue, we subtracted the fluorescence of the negative control (cells harboring the empty plasmid) from that of cells carrying the promoter-reporter construct. Subsequently, we normalized the resulting fluorescence values by the optical density values of cells carrying the plasmid with the promoter area. This artifact diminishes once cell density approaches OD_580_ ≈ 1 (Figure S4C).

An alternative explanation emerges when the promoter in question is transcriptionally active in the stationary phase, in the case of RpoS-dependent promoter PlapF (Figure 7). It may lead to reporter protein accumulation that persists when cells are transferred into fresh medium. As no further synthesis or decreased synthesis of stable reporter protein occurs after culture dilution, fluorescence levels decrease during cell division due to dilution of the preexisting protein pool. This effect is especially pronounced with AAV-tagged reporters, where faster protein turnover accelerates signal decay (Figures 5 and 7).

### Detection of Transcriptional Activation and Repression

To develop a universal reporter system capable of detecting diverse regulatory mechanisms and a wide range of promoter strengths, we modulated fluorescence signal intensity using two strategies: (i) by shortening reporter protein half-life through the incorporation of C-terminal degradation tags, and (ii) by employing plasmids with either low or medium-copy-number origins.

A known limitation of reporter systems is their inability to reliably capture repression dynamics, particularly when the half-life of the reporter protein exceeds the experimental time frame. In such cases, the fluorescence signal persists even after transcription has stopped, as previously synthesized reporter proteins continue to contribute to the signal. This becomes evident in time-course experiments across growth phases as a plateau in fluorescence, where signal intensity no longer changes over time. However, this approach is unsuitable for end point measurements or detection of gene expression in biofilm-associated cells, where only a single signal readout is obtained and no temporal reference is available. To address this, we fused degradation tags to the C-terminus of reporter proteins, specifically, LVA and AAV/ASV tags, to accelerate protein turnover. In *E. coli*, these tags reduce the half-life of GFP to approximately 40 and 60 min, respectively.^34^ In our experiments, we used the PnuoA promoter, which has been reported to be transcriptionally active in both exponential and stationary phases.^33,41,42^ However, even under the control of the relatively strong PnuoA promoter, fluorescence levels from reporters carrying the LVA tag remained low and did not significantly exceed those of the empty vectors (Figure 5). In contrast, constructs with AAV or ASV tags (pA7 and pB8 constructs in Figure 5) provided measurable signal and captured PnuoA-driven expression more reliably than their untagged counterparts (pA3 and pB4 constructs).

We used the reporter system to evaluate transcriptional activation and repression in *P. putida*. This was motivated by the fact that assessing gene expression changes using quantitative PCR (qPCR) is often impractical or extremely challenging. First, qPCR is an invasive technique requiring cell lysis, which makes repeated measurements labor-intensive and introduces artifacts due to temporary changes in growth conditions (e.g., aeration, temperature) and a higher risk of contamination. These factors can lead to unreliable results. Second, qPCR only captures population-level average expression and provides no information about single-cell variability, which is especially critical in structured communities such as biofilms. In contrast, fluorescence-based reporter systems offer faster and less labor-intensive alternatives, and fusion protein-based reporters provide more direct insight into gene expression than mRNA quantification alone. Admittedly, neither method is perfect; reporter plasmids inherently conflate transcription and translation effects. Nevertheless, we were particularly interested in using medium-copy-number plasmids (BBR1 origin) to study transcriptional regulation, as weak or very weak promoters often fail to produce a detectable signal when expressed from low-copy-number plasmids.

Measurement of transcriptional activation was straightforward for both plasmid copy number variants (BBR1 and RK2; Figure S2). Transcriptional repression could also be detected using both plasmid types, provided that the reporters used accumulated in the cell over time (Figures 6 and S3). However, the signal from the low-copy-number RK2-based plasmid pYR7, which encoded reporters with AAV/ASV tags, was too weak to be reliably distinguished from background noise, making repression detection challenging or infeasible in this context (Figures 6 and S3). Moreover, since only direct measurement of mRNA levels would yield absolute quantitative results, reporter systems inherently provide relative values (similarly, RT-qPCR will provide relative values). Thus, the key consideration is whether transcriptional repression can be detected qualitatively (is it detectable or not). Considering the signal strength of the reporter system and whether it provides a strong enough signal for comparative data analysis, we can draw a parallel between the situation of AAV-tagged reporter proteins encoded on RK2 plasmids (pYR7) and low transcription initiation from weak promoters, both of which result in an undetectable signal in the case of low-copy-number plasmids. Based on this, we propose that medium-copy-number plasmids (BBR1) are effective for studying transcription from weak to moderately strong promoters, as they generate a sufficiently strong signal that can detect changes in gene expression or distinguish from background noise. In contrast, low-copy-number plasmids (RK2) are better suited for analyzing strong promoters.

### Application in Sessile Cells and Biofilm Analysis

Since antibiotic diffusion is reduced in biofilms, the plasmid carrying the reporter genes must be stable in cells even without antibiotics. While BBR1 plasmids generally exhibit high stability in *P. putida* even without a toxin-antitoxin system (Figure 2B), we hypothesized that incorporating the *hok-sok* system could further enhance plasmid maintenance, particularly in cases where reporter gene expression imposes a high metabolic burden on the cells. Additionally, we required AAV/ASV-tagged reporter proteins to monitor gene expression reduction in 24-h-old biofilms, ensuring that the observed signal represents *de novo* expression rather than protein synthesized before biofilm formation.

For these experiments, we utilized a fusion promoter area PnuoA-PlapF, which allowed for semiconstitutive expression of a reporter gene from PnuoA and stationary phase-dependent expression from PlapF. This design would allow us to identify conditions when the *lapF* promoter is not active in cells, using PnuoA-driven SYFP2 expression as a control.

However, we encountered challenges in fluorescence microscopy due to the low photostability of Scarlet-I3. As a result, we opted to use the pBVS2–7-AY-FR plasmid (Figure 8A), which encodes Scarlet-I-AAV and mVenus-AAV reporter genes for improved stability and signal detection (Table S3, Figure 8).

It should be emphasized that our objective was not to assess *lapF* expression in biofilms, but rather to assess the possibility of studying expression dynamics in biofilm layers. First, we constructed an artificial promoter region, PnuoA-PlapF. PnuoA should constantly initiate transcription of the reporter gene (mVenus-AAV) in cells, but as the transcription from PlapF is dependent on RpoS, the reporter gene (Scarlet-I-AAV) may not be expressed uniformly across the biofilm. Since we are comparing the signal of two reporters, cells that have lost the plasmid do not affect the assessment of expression dynamics, because we are comparing the activity of two promoters in the biofilm with each other. On the other hand, such artificially fused promoter area may not accurately reflect *lapF* expression, because to assess *lapF* expression, it is necessary to use a promoter region that is native to the *P. putida* chromosome.

However, the created reporter system is ideal for comparing the expression of two adjacent and oppositely oriented genes, whose transcriptions are initiated from the same promoter area, but in opposite directions. The shorter the promoter region between two opposite genes, the more likely it is that the expression regulations of the genes influence each other, and their expression may be different for biofilm-related genes in sessile cells. Thus, the mVenus-Scarlet-I-based reporter systems allow us to detect the expression dynamics of two adjacent but oppositely orientated genes in sessile cells and enable us to analyze the biofilm layers (Figure 8).

### Possibility of Excludon Mechanism in *P. putida*

Evaluation of the potential leakiness of inducible promoters yielded two surprising findings. Based on transcriptional regulation mechanisms, we expected a significant increase in fluorescence in cells harboring pC13A-AAV, which contains the Scarlet-I3-AAV gene under the control of the Ptac promoter. Comparing the fluorescence of cells with pC13A-AAV grown in LB medium without IPTG supplementation to the cells with pYR7, only slightly higher fluorescence was observed in favor of pC13A-AAV (Figure S4). Thus, the Ptac promoter contributes negligibly to the reporter signal in the absence of induction and is relatively well controlled by LacI.

In contrast to the tightly regulated Ptac promoter, *Pm* exhibited substantial leakiness. In our construct, the *Pm* promoter was positioned downstream of the Scarlet-I3-AAV gene, causing the transcription of asRNA complementary to the reporter mRNA, which is not directly measurable by fluorescence. However, RT-qPCR analysis revealed detectable levels of asRNA even in the absence of the inducer m-toluate, which is required for transcriptional activation from *Pm* (Figure 9E), indicating leaky transcription. Surprisingly, asRNA was not detectable in cells exposed only to m-toluate, whereas it was consistently observed in cells also producing Scarlet-I3-AAV mRNA by IPTG (Figure 9E). A plausible explanation is that, in the presence of mRNA, asRNA forms a duplex with its target transcript, thereby stabilizing the otherwise rapidly degraded antisense RNA, which lacks ribosomal protection. In the absence of target mRNA, unpaired asRNA may be quickly degraded and fall below the detection limit.

If duplex formation were to occur, it would support an excludon-based mechanism of translation inhibition for Scarlet-I3-AAV. In such a model, the mRNA–asRNA duplex obstructs translation, which is consistent with the observed decrease in cellular fluorescence upon coinduction with IPTG and m-toluate compared to the fluorescence when only IPTG was added to the medium (Figures 9B,C, S4C). However, we cannot exclude the possibility that the reduced cell fluorescence upon coinduction with IPTG and m-toluate is caused by the collision of RNA-polymerases. We used primers for RT-qPCR that bind the middle of the Scarlet-I3-AAV gene, and the amount of RNA may not be the same as the amount of RNA being translated. It is possible that the elongation of RNA transcription was terminated as a result of collisions further away from the amplified region, and we detected a larger amount of mRNA than was actually being translated.

Interestingly, strong reporter gene expression in cells harboring pC13A-AAV was observed only up to the early stationary phase, followed by a pronounced decline, despite the presence of 0.5 mM IPTG in the medium (Figure S4C). This decline is likely attributable to a physiological response of the bacterial cells, wherein global transcriptional activity is reduced during the transition to the stationary phase due to energy limitations. Notably, this decrease in fluorescence illustrates the utility of the reporter system in detecting repression of gene expression. However, the reporter signal must be sufficiently strong, whether due to high transcriptional activity (as in the case of Ptac induction by IPTG) or elevated reporter gene copy number (in the case of weak promoters), for such repression to be reliably detected.

In principle, it is possible to detect the presence of excludon in the promoter areas of divergently oriented promoters using the created reporter plasmids. To ascertain this, it is necessary to compare the fluorescence of cells and quantify the reporter gene mRNA. Therefore, two types of promoter areas are necessary to insert into the reporter plasmid: a native promoter area and a promoter area carrying promoters that function only in one direction, with the promoters in the opposite direction being mutated. If the amount of mRNA of the gene under study (the corresponding reporter gene) remains unchanged, but in the case of the mutated construct, the fluorescence of the cells is enhanced, then this indicates regulation of gene expression by excludon.

## Conclusions

We have identified suitable fluorescent reporter proteins that enable rapid and efficient assessment of promoter areas with promoters in two directions. This reporter system is developed for *P. putida* to assess gene expression directly from complex growth media such as LB. We constructed reporter systems to study transcription driven by both weak and strong promoters. Furthermore, we demonstrated that the developed reporter system is useful for assessing gene expression in conditions requiring noninvasive methodologies, such as detecting expression in biofilms, or in situations requiring the detection of more complex regulatory mechanisms, such as gene expression by excludon, RNAP–RNAP or RNAP-DNAP collision.

## Material and Methods

### Bacterial Strains and Culture Conditions

The wild-type *P. putida* strain PaW85, which differs from KT2440 by 49 point mutations, mostly located in intergenic regions, and a *fis*-overexpression derivative, strain F15 (Gm^r^), were used in this study.^43–45^ Bacteria were cultivated in either lysogeny broth (LB) medium or M9 minimal medium.^7^ M9-based media were supplemented with 2.5 mL/L microelement solution,^46^ 2 g/L d-glucose, and when necessary, 2 g/L cas-amino acids (Difco CAA, Gibco, Thermo Fisher Scientific, Waltham, MA) along with 0.01 g/L tryptophan.

Cultures were grown at 30 °C, shaking at 180 rpm, with or without antibiotic supplementation. Antibiotics used for plasmid maintenance included kanamycin (50 μg/mL), penicillin G (1 mg/mL in liquid media or 1.5 mg/mL on solid media), and gentamicin (10 μg/mL) for selection of *P. putida* F15 during precultivation. For biofilm assays, antibiotics were omitted. Overexpression of *fis* in strain F15 was induced using 0.5 mM IPTG for the induction of transcription of a Ptac promoter.

For excludon evaluation, LB medium was used as the growth medium, and IPTG or m-toluate were added as effectors at final concentrations of 0.125 mM, 0.25 mM, or 0.5 mM. Negative controls were grown without effectors.

The protocol for fluorescence assessment was optimized before experiments. Colonies recovered after electroporation were plated in sectors, and after overnight growth, they were used for inoculation of media (2-day-old cells). For end point measurements, cells were grown in 5 mL of media for 18 h at 30 °C with shaking (180 rpm), after which absorbance and fluorescence were recorded. The end point measurements were repeated at least three times. For continuous assay, four different colonies from two separate electroporations were used for overnight cultivation in 5 mL of media with appropriate antibiotics. The next day, the overnight cultures were diluted to an initial OD580 of 0.2 in 200 μL of fresh medium and incubated in Greiner 96-well μClear microtiter plates (item no. 655090; Kremsmünster, Austria) for 22–24 h at 30 °C with orbital shaking (365 cpm).

For biofilm assays, cells were precultivated as described above for end point measurements, then 3 mL of antibiotic-free medium was inoculated and incubated for 24 h at 30 °C in microscopy-compatible chambers μ-Slide 8 Wellhigh Glass Bottom (item no 80807; ibidi USA, Inc., Fitchburg, WI) without shaking.

### Plasmid Construction

Cloning was performed using restriction enzyme digestion and ligation with T4 DNA ligase or fusion-PCR. Restriction, ligations, and PCR amplifications were conducted according to the manufacturer’s instructions (Thermo Fisher Scientific, Vilnius, Lithuania). In fusion-PCR, DNA fragments were amplified with specific primers and fused without primers in a subsequent reaction, as previously described.^47^ PCR and restriction-generated fragments were separated on 1% (w/v) agarose gels and purified using the GeneJET Gel Extraction Kit (Thermo Fisher Scientific, Vilnius, Lithuania).

PCR, restriction digestion, and Sanger sequencing verified all constructs. The design principles and resulting plasmids are listed in Table S3. Plasmids were introduced into *P. putida* via electroporation at 2500 V using the competent cells in saccharose as a previously described method.^48^

Synthetic DNA was designed under the following criteria: (i) no promoter sequences with a higher score (LDF) than 3 in direction to the gene 5′ end as predicted by BPROM (www.softberry.com, Softberry, Inc., Mount Kisco, NY); (ii) restriction sites (e.g., *Bam*HI, Acc65I, XbaI) were included only in the positions for cloning; (iii) no transcriptional terminators were present within the coding region; (iv) codon usage of *Escherichia coli* in VectorBuilder (www.vectorbuilder.com, Vectorbuilder Inc., Chicago, IL) was used as a similariest Gram-negative bacterium to *P. putida*, which codon usage was available, and SnapGene v8.1 (Dotmatics, San Diego, CA). Synthetic DNAs were synthesized by Twist Bioscience (San Francisco, CA).

A plasmid was constructed based on a dynamic design that was iteratively modified throughout the study in response to experimental results and research objectives. The key design principles were as follows: (i) constructing a bidirectional reporter cassette, the reporter genes were positioned in opposite orientations within the reporter cassette to allow for simultaneous assessment of divergent transcription. (ii) Minimization of promoters, to prevent interference with gene expression analysis, putative internal promoters within the reporter genes were reduced by designing synthetic DNA sequences (stDNAs) that minimized promoter-like elements. (iii) Optimized ribosome binding sites (RBS), an RBS was placed 5′ of each reporter gene, with sequence variations introduced to prevent unintended mRNA hybridization and secondary structure formation. (iv) Reporter gene variants for fusion protein construction, parallel plasmid versions were engineered with reporter genes lacking a start codon and RBS, enabling fusion protein construction between the reporter and the gene of interest. (v) Cloning site standardization, a single *Bam*HI restriction site was introduced between the reporter genes for promoter region insertion, thereby minimizing sequence-based expression variability. (vi) Reduced reporter protein stability, degradation tags were incorporated into the reporter proteins to decrease their half-life, enhancing the temporal resolution of gene expression changes. (vii) Transcriptional isolation, transcription terminators flanked the reporter cassette to prevent activation by extraneous promoters outside the cassette, reducing unintended transcription. (viii) The ampicillin-resistance gene was chosen as the primary selection marker to exclude direct antibiotic effects on transcription and translation. Alternative antibiotic resistance markers were used to evaluate fluorescent protein suitability in *P. putida*. (ix) The toxin-antitoxin system for plasmid maintenance and the *hok-sok* system were incorporated to facilitate plasmid maintenance in cells in antibiotic-free environments. (x) Plasmid copy number variability, due to differences in promoter strength, the reporter system was constructed using two plasmid backbones: RK2-type plasmids (low-copy number) and BBR1-type plasmids (medium-copy number). Tables S2 and S3 summarize the constructed plasmids and the DNA elements necessary for the reporter system.

### The Assessment of the Burden of Plasmids on *P. putida* and Plasmid Persistence in *P. putida*

The burden of plasmids on bacteria was assessed by colony area. For this purpose, cells were grown for 24 h in 5 mL LB medium with the appropriate antibiotic. Then, the cell culture was diluted so that approximately 1 × 102 to 2 × 102 colonies would form on the LB plate supplemented with antibiotics. Bacteria were grown for 24 h, and then the colony area was measured using the program UVP software RC1.2 Version 8.0 (VisionWorks LS, Cambridge, UK).

Repeated subcultures assessed plasmid persistence in cells. For this purpose, cells were grown overnight in 5 mL LB with the appropriate antibiotic. Then, 5 mL LB without antibiotics was inoculated with 100 μL of cell culture, and bacteria were cultivated for 6–8 h. Growth cycles without antibiotics were repeated 7 times. In the last cycle, cells were allowed to grow overnight, and 10-fold dilutions were plated on LB with and without antibiotics and then grown overnight (approximately 45 generations in total with all 8 reinoculations). Plasmid persistence was assessed by the number of colonies on both plates the following day. Both experiments were repeated three times.

### Assessment of Fluorescence

Fluorescence and absorbance (580 nm) were assessed by end point or continuous measurements in microtiter plates. Measurements were performed using an Agilent BioTek Synergy H1 microplate reader (Santa Clara, CA) with Greiner 96-well μClear plates (item no. 655090; Kremsmünster, Austria). Fluorescence was measured at a gain of 50 using appropriate excitation/emission wavelengths for each fluorescent protein (Table S1). When dual-reporter constructs were used, the fluorescence of the reporter protein with the longer emission wavelength was assessed first.

For end point measurements, 150 μL of culture was transferred to wells directly. In continuous measurements, each well contained 200 μL of culture, and fluorescence and absorbance were recorded every 20 min throughout the incubation.

Measurements were taken at the excitation and emission wavelengths corresponding to potential fluorescent reporter proteins: TagBFP (BFP): Ex 381 nm (±20), Em 450 nm (±20), mCerulean (CFP): Ex 433 nm (±20), Em 476 nm (±20), Gfpmut2 (GFP): Ex 488 nm (±20), Em 510 nm (±20), mVenus in pBLKT_VS: Ex 505 nm (±20), Em 538 nm (±20), mVenus in pBLKT_VS2­(T) and SYFP2 in pA3: Ex 505 nm (±10), Em 540 nm (±10), Scarlet-I and Scarlet-I3: Ex 570 nm (±20), Em 600 nm (±20).

The fluorescence of bacteria for end point assays was shown as fluorescence units without normalization (FU) or as relative fluorescence units (RFU) by normalizing fluorescence to the optical density (580 nm) of the culture. In continuous assays, the fluorescence of cells carrying an empty vector (no promoter upstream of reporter genes) was subtracted from experimental values, and the resulting fluorescence was normalized to the optical density (580 nm) of the culture carrying plasmids with promoters in front of reporter genes and indicated in figures as RFU ((Em1-Em0)/OD1).

### Quantification of RNA by RT-qPCR

Cells grown overnight were subcultured into fresh 5 mL LB + 1000 μg/mL penicillin medium and grown for 18 h to study gene expression dependent on the reporter cassette orientation; and to exponential phase (3 h) to study the excludon mechanism, thereafter 0.5 mM IPTG and/or 0.5 mM m-toluate were added and grown with effectors for another 6 h (no effectors were added to the negative control).

RNA was isolated using NucleoSpin RNA Kit (Macherey-Nagel, Düren, Germany) following the manufacturer’s protocol. Afterward, isolated RNA was treated with Dnase I to remove DNA contamination (Thermo Fisher Scientific, Vilnius, Lithuania). RNA concentration and purity were assessed using a NanoDrop ND-1000 spectrophotometer (Thermo Fisher Scientific, Waltham, MA). RNA integrity was assessed by running agarose gel electrophoresis (2% (w/v) agarose gel, 100 V for 20 min, followed by staining with ethidium bromide). RT-qPCR was performed in a RotorgeneQ 5-plex HRM platform (Qiagen GmbH, Hilden, Germany). Negative (nuclease-free water) and no-reverse transcriptase controls were included in each run. Melting curve analysis was performed after a run to validate primers. All used oligonucleotides are shown in Table S4.

For the experiment where we tested the reporter cassette orientation effect, one-step qPCR analysis was performed using SOLIScript 1-step SolisGreen Kit 2.0 (Solis BioDyne, Tartu, Estonia) as per the manufacturer’s instructions. Data from three separate RT-qPCR experiments performed on three independently extracted RNAs were averaged and normalized against *rpoD* levels.

For the excludon mechanism experiment, we used two-step qPCR for the amplification of sense and antisense strands of the transcript. To generate a complementary strand of cDNA from RNA, reverse transcription of 1.5 μg RNA was performed with Maxima H Minus Reverse Transcriptase (Thermo Fisher Scientific, Vilnius, Lithuania) following the manufacturer’s instructions. Subsequently, cDNA was purified with the ZymoResearch DNA Clean and Concentrator-5 kit (Zymo Research, Irvine, CA). RT-qPCR was performed using the Maxima SYBR Green qPCR Master Mix (2X) (Thermo Fisher Scientific, Vilnius, Lithuania) by the manufacturer’s protocol. Data from three separate qRT-PCR experiments performed on three independently extracted RNAs were averaged and normalized against *polA* levels.

Normalized data were exported from the qPCR machine and imported into LinRegPCR software (version 2021.2)^49^ which was used to analyze qPCR data.

For the reporter cassette orientation experiment, relative expression was calculated using the ΔΔCq method.^50^ Expression ratios were determined between plasmid constructs that differed in cassette orientation but shared the same promoter direction and plasmid backbone (e.g., pB-B4-nuoA-R relative to pB-A3-nuoA-R; pB-A3-nuoA-Y relative to pB-B4-nuoA-Y) to assess differences associated with cassette orientation.

For the C13A-AAV experiment, the samples fold change relative to the untreated control was reported according to the Pfaffl method^51^ (ratio = *E*_target_^ΔCq target (control–sample)^/*E*_reference_^ΔCq reference (control–sample)^). In the program settings, three amplicon groups were defined, one for the sense strand, one for the antisense and one for the housekeeping gene to determine individual amplification efficiencies. The LinRegPCR program was then used to determine the fluorescence threshold for all samples and calculate the individual *C*_q_ and efficiency values.

### Microscopy and Biofilm Imaging

Confocal scanning laser microscopy was used for visualization of the *lapF* and *nuoA* expression within the biofilm. *P. putida* PaW85 harboring pBVS2–7-AY-FR was cultured in Ibidi 8-well coverglass chamber slides under static conditions at 30 °C for 24 h to allow biofilm formation. Each well contained 200 μL of culture. To remove planktonic cells prior to imaging, the existing medium was carefully removed and replaced with fresh medium. Biofilms were imaged using an Axio ObserverZ1/7 confocal laser scanning microscope (Carl Zeiss, Berlin, Germany) with a Plan-Apochromat × 63/1.4 oil objective. Scarlet-I was excited using a 561 nm laser line (5% laser power), and the emission was detected at 574–720 nm. mVenus was excited at 514 nm (2%), and emission was detected between 520 and 562 nm. Z-stack images were acquired at a step of 0.5 μm, for a total of 70 μm, starting from the bottom layer. Image acquisition was performed using the ZEISS ZEN 3.11 (lite version) software. ImageJ software (version 1.54p) was used to calculate relative fluorescence intensity in biofilm layers.^52^

The image analysis protocol was the following: z-stack images were split into individual channel stacks corresponding to the two FP-s. For each layer, the mean fluorescence intensity was calculated. This was done independently for both channels across all slices. The images had 512 × 512 pixels, making a total scanned area of 67.34 × 67.34 μm. Values were normalized, and the mean fluorescence intensity of the slice was divided by the maximum fluorescence observed in the channel. The mean fluorescence intensities of individual cells were determined using the average intensity projections.

## Statistical Analysis

Statistical analyses were performed using TIBCO Statistica (v14.0.1.25; San Ramon). One-way or multifactorial analysis of variance (ANOVA) was conducted where appropriate, followed by Bonferroni posthoc tests (α = 0.05) to assess pairwise differences. In transcriptional repression studies, a General Linear Model (GLM) was applied for time-course fluorescence data to evaluate the effects of time, construct, effectors, and their interactions. Data from 15 h onward were included. In excludon experiments, data from 3 h onward were included. In some cases, statistical analysis was not performed as the experiment was only for quantitative visualization, and the statistical analysis was not needed. Homogeneous groups resulting from the Bonferroni tests are indicated in the figures using letter annotations. The same letter above columns indicates no significant difference between groups (*p* ≥ 0.05).

## Supplementary Material











## Notes

The fluorescence of *P. putida* PaW85 without plasmids and with empty plasmids (Figure S1); evaluation of transcriptional activation (Figure S2); evaluation of transcriptional repression (Figure S3); the background fluorescence of *P. putida* PaW85 harboring the reporter plasmids pYR7 and pC13A-AAV (Figure S4) (PDF)
Fluorescent proteins used in the work (Table S1) (PDF)
DNA elements in reporter gene cassettes, hypothetical promoters, terminators and RBS binding sequences of constructed plasmids (Table S2) (PDF)
Used and constructed plasmids (Table S3) (PDF)
Used oligonucleotides for RT-qPCR (Table S4) (PDF)
