# Supplementary material for: A Reporter System for Assessment of Transcription from Divergently Oriented Promoters in Pseudomonas putida
Source: ACS Synth Biol. 2025 Dec 10;14(12):4947–66. doi: 10.1021/acssynbio.5c00723 (PMC12723746; doi:10.1021/acssynbio.5c00723)
Supplement: Supplementary file 1 [file sb5c00723_si_001.pdf]

Supporting Information for Publication

A reporter system for assessment of transcription from divergently oriented promoters in *Pseudomonas putida*

Johanna Hendrikson, Mia-Lota Keskküla, Gea M. Räis, Maia Kivisaar and Riho Teras*

Institute of Molecular and Cell Biology, University of Tartu, 51010 Estonia

* Corresponding author, riho.teras@ut.ee

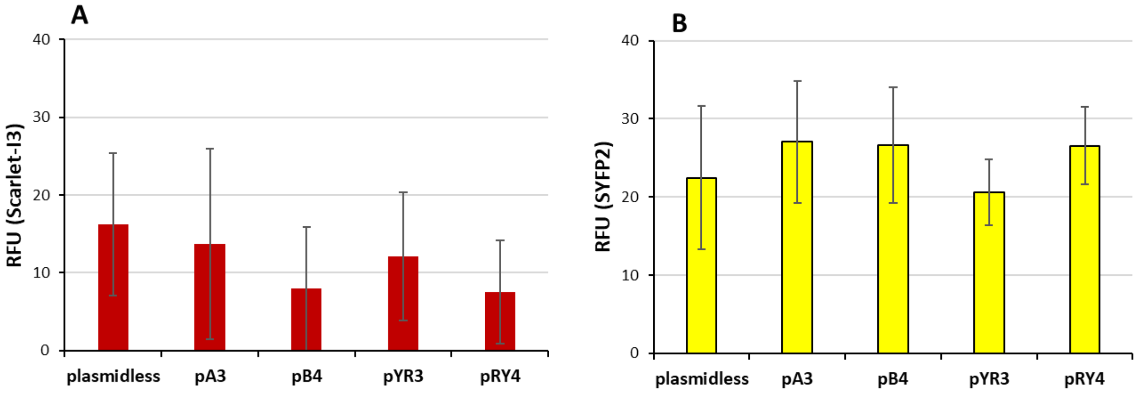

Figure S1. The fluorescence of P. putida PaW85 was grown in LB for 18 hours without plasmids and with empty pA3, pB4, pYR3 or pRY4 plasmids. (A) Scarlet-I3 (ex 570 nm (20), em 600 nm (20)) and (B) SYFP (ex 505 nm (10), em 540 nm (10) relative fluorescence. Displayed values represent the arithmetic mean, with error bars indicating the 95% confidence interval. Statistical analysis was performed using one-way ANOVA (n=9).

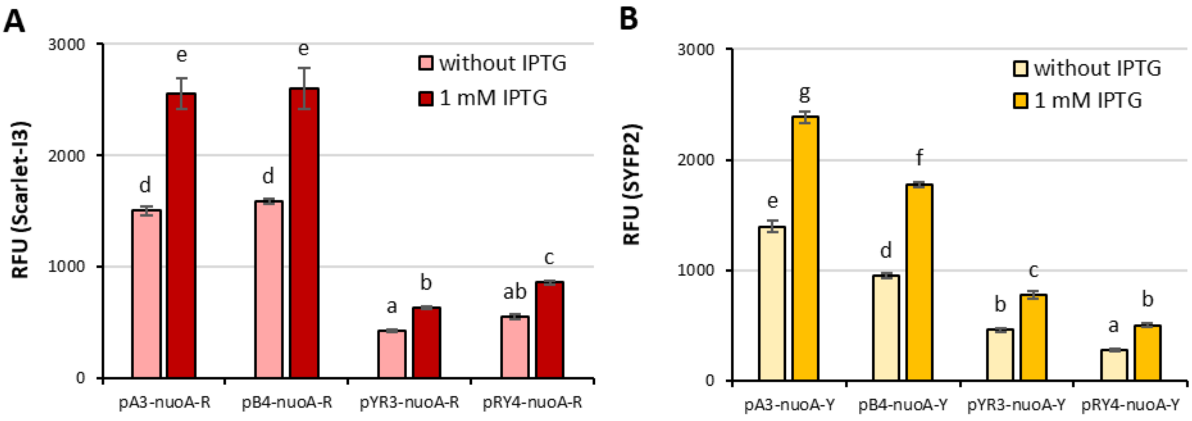

Figure S2. Evaluation of transcriptional activation using the developed reporter systems in P. putida. Cells were grown in LB medium supplemented with 1000 µg/ml penicillin G for 18 hours. Cultures were incubated either with or without 1 mM IPTG. (A) Relative fluorescence of Scarlet-I3 (ex 570 nm (20), em 600 nm (20)) in cells carrying pA3-nuoA-R, pB4-nuoA-R, pYR3-nuoA-R, or pRY4-nuoA-R. (B) Relative fluorescence of SYFP2 (ex 505 nm (10), em 540 nm (10)) in cells carrying pA3-nuoA-Y, pB4-nuoA-Y, pYR3-nuoA-Y, or pRY4-nuoA-Y. Displayed values represent the arithmetic mean, with error bars indicating the 95% confidence interval. Multifactorial ANOVA was calculated from log10 values, with homogeneity groups indicated above the bars (n=4).

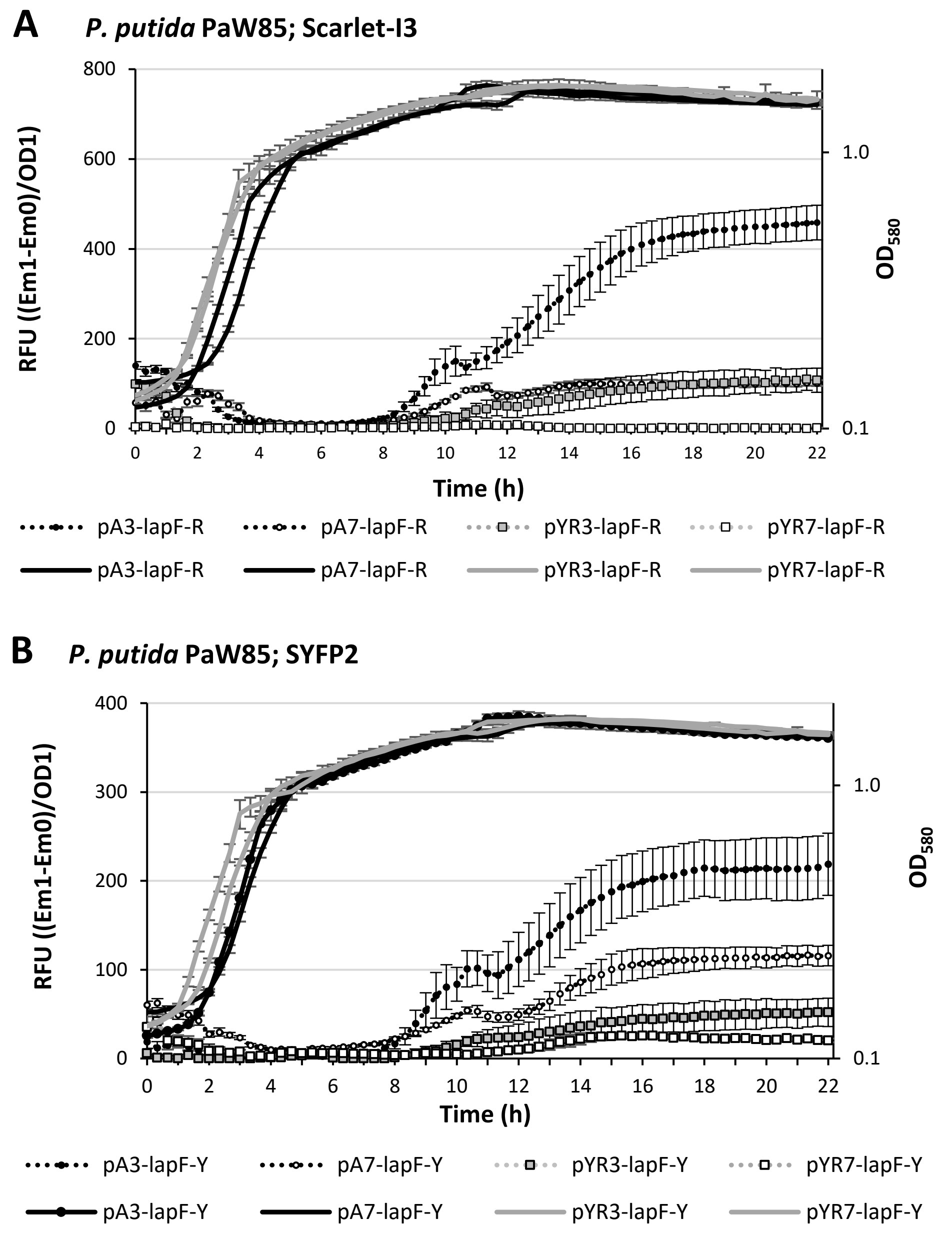

Figure S3. Evaluation of transcriptional repression using the developed reporter systems in P. putida. P. putida PaW85 carrying the respective plasmids were cultivated for 22 hours in LB medium supplemented with 1000 µg/ml penicillin G in a microtiter plate. Optical density at 580 nm and fluorescence of (A) Scarlet-I3 (ex 570 nm (20), em 600 nm (20)) and (B) SYFP (ex 505 nm (10), em 540 nm (10)) were measured every 20 minutes. Solid lines without markers represent growth curves, while fluorescence is shown as dashed lines. The fluorescence of the following constructs is indicated: pA3-lapF-R/Y (black dashed line, black circles), pA7-lapF-R/Y (black dashed line, open circles), pYR3-lapF-R/Y (grey dashed line, grey squares), and pYR7-lapF-R/Y (grey dashed line, open squares).

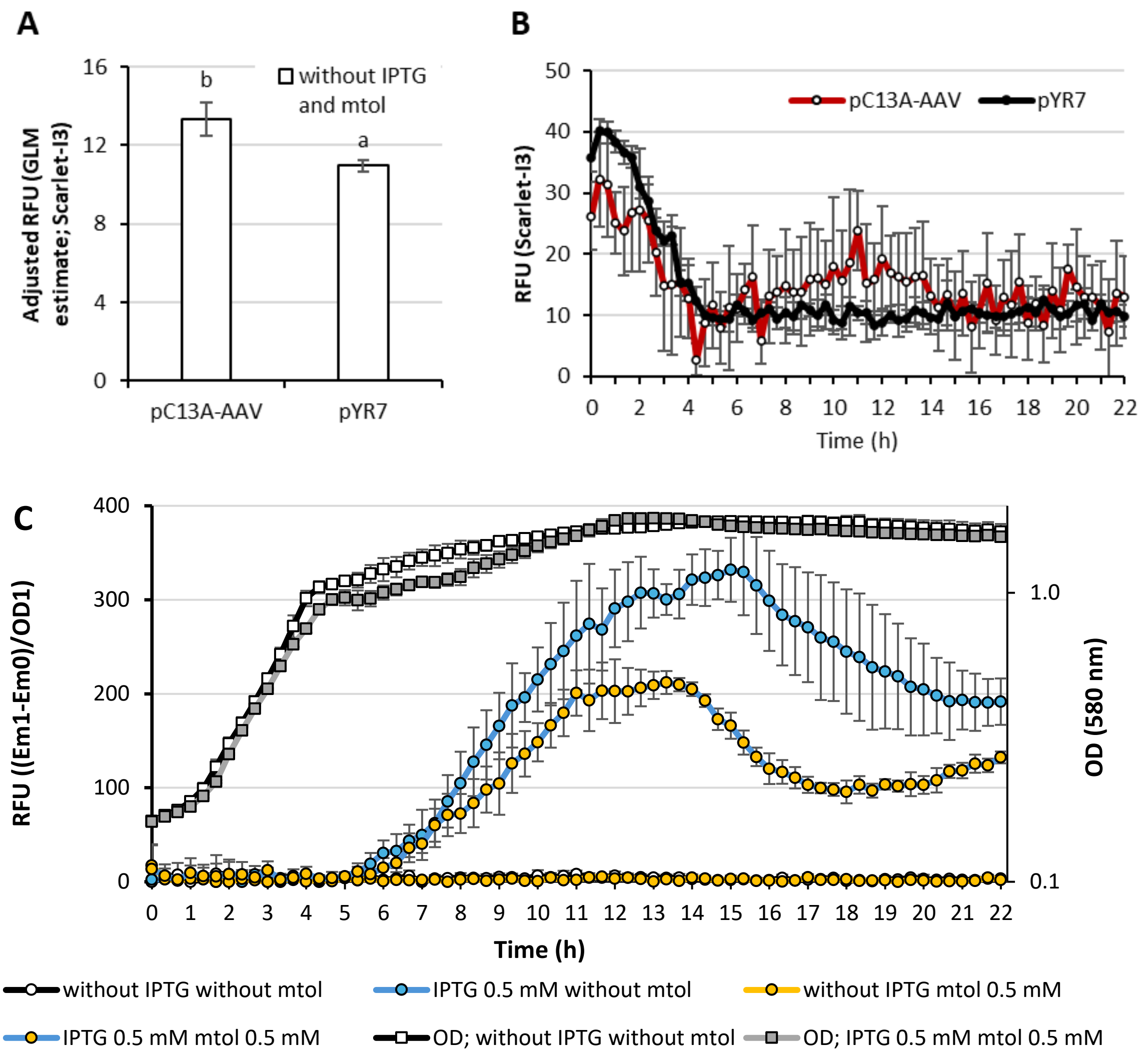

Figure S4. The background fluorescence of P. putida PaW85 harbouring the reporter plasmids. Cells harbouring test plasmids (schemes are indicated in Figure 9) were cultivated in LB medium supplemented with 1000 µg/ml penicillin G in a 96-well microtiter plate without effectors. Fluorescence intensity of cells (570 (±20), 600 (±20)) was measured every 20 minutes. (A) The data from 3 hours to 22 hours were analysed using the General Linear Model (GLM), and the adjusted relative fluorescence values are presented in the figure. (B) The relative fluorescence units (RFU) of the cells were measured for 22 hours of growth in media without effectors. (C) The relative fluorescence units (RFU) of P. putida PaW85 harbouring pC13A-AAV grown in LB containing four combinations of effectors are shown: no effectors added; 0.5 mM IPTG added; 0.5 mM m-toluate added; and both effectors added (0.5 mM each). IPTG induces reporter gene mRNA synthesis, and m-toluate induces asRNA synthesis. Two growth curves are also shown: one for cultures grown without effectors and one for cultures grown with both effectors added (0.5 mM each). The error bars indicate the 95% confidence interval.
