# Supplementary material for: A Reporter System for Assessment of Transcription from Divergently Oriented Promoters in Pseudomonas putida
Source: ACS Synth Biol. 2025 Dec 10;14(12):4947–66. doi: 10.1021/acssynbio.5c00723 (PMC12723746; doi:10.1021/acssynbio.5c00723)
Supplement: Supplementary file 2 [file sb5c00723_si_002.pdf]

Supporting Information for Publication

A reporter system for assessment of transcription from divergently oriented promoters in *Pseudomonas putida*

Johanna Hendrikson, Mia-Lota Keskküla, Gea M. Räis, Maia Kivisaar and Riho Teras*

Institute of Molecular and Cell Biology, University of Tartu, 51010 Estonia

* Corresponding author, riho.teras@ut.ee

Table S1. Fluorescent proteins used in the work and their most important characteristics

Fluorescent protein (oligomerization)[1]	Used wavelength for excitation (optimal wavelength)	Used wavelength for emission (optimal wavelength)	Maturation time	Brightness (B) and quantum yield (QY)
TagBFP [1] (monomer)	381 nm ±20 (402 nm)	450 nm ±20 (457 nm)	13.0 min	B: 32.76<br>QY: 0.63
mCerulean [2]<br>(monomer)	433 nm ±20 (433 nm)	475 nm ±20 (475 nm)	6.6 min	B:16.17<br>QY: 0.49
Gfpmut2 [3]<br>(weak dimer)	488 nm ±20 (485 nm)	510 nm ±20 (508 nm)	5.6 min	B: 39.35<br>QY: 0.73
mVenus [4]<br>(monomer)	505 nm ±20 (515 nm) or<br>505 nm ±10 (515 nm)	538 nm ±20 (527 nm) or<br>540 nm ±10 (527 nm)	17.6 min	B: 66.56<br>QY: 0.64
SYFP2 [4]<br>(monomer)	505 nm ±10 (515 nm)	540 nm ±10 (527 nm)	4.1 min	B: 68.68<br>QY: 0.68
Scarlet-I [5]<br>(monomer)	570 nm ±20 (569 nm)	600 nm ±20 (593 nm)	36.0 min	B: 56.16<br>QY: 0.54
Scarlet-I3 [6]<br>(monomer)	570 nm ±20 (568 nm)	600 nm ±20 (592 nm)	2.0 min	B: 68.25<br>QY: 0.65

[1] Data obtained from FP database [7]

1. Subach, O. M.; Gundorov, I. S.; Yoshimura, M.; Subach, F. V.; Zhang, J.; Gruenwald, D.; Souslova, E. A.; Chudakov, D. M.; Verkhusha, V. V., Conversion of red fluorescent protein into a bright blue probe. *Chemistry & biology* **2008,** *15* (10), 1116-24.
2. Rizzo, M. A.; Piston, D. W., High-contrast imaging of fluorescent protein FRET by fluorescence polarization microscopy. *Biophysical journal* **2005,** *88* (2), L14-6.
3. Cormack, B. P.; Valdivia, R. H.; Falkow, S., FACS-optimized mutants of the green fluorescent protein (GFP). *Gene* **1996,** *173* (1 Spec No), 33-8.
4. Kremers, G. J.; Goedhart, J.; van Munster, E. B.; Gadella, T. W., Jr., Cyan and yellow super fluorescent proteins with improved brightness, protein folding, and FRET Forster radius. *Biochemistry* **2006,** *45* (21), 6570-80.

5. Bindels, D. S.; Haarbosch, L.; van Weeren, L.; Postma, M.; Wiese, K. E.; Mastop, M.; Aumonier, S.; Gotthard, G.; Royant, A.; Hink, M. A.; Gadella, T. W., Jr., mScarlet: a bright monomeric red fluorescent protein for cellular imaging. *Nature methods* **2017,** *14* (1), 53-56.
6. Gadella, T. W. J., Jr.; van Weeren, L.; Stouthamer, J.; Hink, M. A.; Wolters, A. H. G.; Giepmans, B. N. G.; Aumonier, S.; Dupuy, J.; Royant, A., mScarlet3: a brilliant and fast-maturing red fluorescent protein. *Nature methods* **2023,** *20* (4), 541-545.
7. Lambert, T. J., FPbase: a community-editable fluorescent protein database. *Nature methods* **2019,** *16* (4), 277-278.
