# Supplementary material for: A Reporter System for Assessment of Transcription from Divergently Oriented Promoters in Pseudomonas putida
Source: ACS Synth Biol. 2025 Dec 10;14(12):4947–66. doi: 10.1021/acssynbio.5c00723 (PMC12723746; doi:10.1021/acssynbio.5c00723)
Supplement: Supplementary file 3 [file sb5c00723_si_003.pdf]

Supporting Information for Publication

A reporter system for assessment of transcription from divergently oriented promoters in *Pseudomonas putida*

Johanna Hendrikson, Mia-Lota Keskküla, Gea M. Räis, Maia Kivisaar and Riho Teras*

Institute of Molecular and Cell Biology, University of Tartu, 51010 Estonia

* Corresponding author, riho.teras@ut.ee

Table S2. DNA elements in reporter gene cassettes, hypothetical promoters, terminators and RBS binding sequences of constructed plasmids

Construct; Length of reporter cassette for analysis[1]	Reporter gene	Predicted promoters Score for promoter (LDF)*; the position from ATG; predicted boxes and boxes Score[2]	The sequence of the hypothetical promoter Consensus of RpoD-type promoters TTGACA $N_{17\pm1}$ TATAAT[3]	Terminators[4]	The sequence in front of the gene, RBS in bold
pBLKT-cfp-gfpV2 1586 bp	In the front of *gfp*	4.26; -477…-510 bp -10 box CATTATATT Score 55 -35 box GTGATA Score 25	GTGATA N_{22} TATATT	rrnB T1; rrnB T2	5' AGGAGGAAAAACAT
	In the front of *cfp*	2.12; -633 …-693 bp -10 box GTTTAGACT Score 56 -35 box TTTTCG Score 28	TTTTCG N_{19} TAGACT	Lambda t0	5' AGGAGGAGGAAA
pBLKT_cfp_yfp (mVenus) 1586 bp	In the front of *yfp*	4.26; -477…-510 bp -10 box CATTATATT Score 55 -35 box GTGATA Score 25	GTGATA N_{22} TATATT	rrnB T1; rrnB T2	5' AGGAGGAAAAACAT
	In the front of *cfp*	2.72; -366…-390 bp -10 box TTTTATAGT Score 62	TTAACT N_{13} TATAGT	his term; RNAI term; TL17; TR2-17	5' AGGAGGAGGAAA

		-35 box TTAACT Score 38			
		2.00; -766…-792 bp<br>-10 box CTGCATTAT Score 43<br>-35 box TTCTCG Score 27	TTCTCG N_{15} CATTAT		
pBLKT_bfp_yfp (mVenus)<br>1617 bp	In the front of *yfp*	5.36; -430…-458 bp<br>-10 box TGGTAAAGT Score 63<br>-35 box TTGCAT Score 50	TTGCAT N_{17} TAAAGT	rrnB T1; rpsI term	5' AGGAGGAAAAACAT
		2.07; -45…-73 bp<br>-10 box ATGTATACC Score 34<br>-35 box TTGATC Score 36	TTGATC N_{17} TATACC		
	In the front of the mTagBFP2 gene	2.72; -367…-391 bp<br>-10 box TTTTATAGT Score 62<br>-35 box TTAACT Score 38	TTAACT N_{13} TATAGT	his term; RNAI term; TL17; TR2-17	5' AGGAGGTATACAT
		2.00; -767…-793 bp<br>-10 box CTGCATTAT Score 43<br>-35 box TTCTCG Score 27	TTCTCG N_{15} CATTAT		
pBLKT-VS<br>1573 bp	In the front of the Scar-let-I gene	2.30; -771…-801 bp<br>-10 box GGCTTTTAT Score 33<br>-35 box TTGCTA Score 53	TTGCTA N_{19} TTTTAT	rrnB T1; rpsI term	5' AGGAGGAAAAACAT
	In the front of the mVe-nus	2.45; -55…-83 bp<br>-10 box TGACACCAT Score 22<br>-35 box TTGATC Score 36	TTGATC N_{17} CACCAT	his term; RNAI term; TL17	5' AGGAGGAATTAAGC
pBLKT-VS2(T)<br>1569 bp	In the front of the Scar-let-I gene	2.72; -367…-391 bp<br>-10 box TTTTATAGT Score 62<br>-35 box TTAACT Score 38	TTAACT N_{13} TATAGT	rrnB T1; rpsI term	5' AGGAGGAAAAACAT

	In the front of the mVenus	2.21; -35…-64 bp<br>-10 box TCCTAAACA Score 19<br>-35 box TTGCTC Score 31	TTGCTC N_{18} TAAACA	his term; RNAI term	5' AGGAGGAGGAAAA
pA3<br>1564 bp	In the front of the Scarlet-I3 gene	2.76; -371…-395 bp<br>-10 box TTTTATAGT Score 62<br>-35 box TTCACT Score 41	TTCACT N_{13} TATAGT	rrnB T1; rpsI term	5' AGGAGGAAAAACAT
	In the front of the SYPF2	2.57; -560…-587 bp<br>-10 box GGCCACCAT Score 25<br>-35 box TTGAAA Score 60	TTGAAA N_{16} CACCAT	his term; RNAI term	5' AGGAGGATGGAAAA
pB4<br>1564 bp	In the front of the Scarlet-I3 gene	2.76; -371…-395 bp<br>-10 box TTTTATAGT Score 62<br>-35 box TTCACT Score 41	TTCACT N_{13} TATAGT	his term; RNAI term	5' AGGAGGAAAAACAT
	In the front of the SYPF2	2.57; -560…-587 bp<br>-10 box GGCCACCAT Score 25<br>-35 box TTGAAA Score 60	TTGAAA N_{16} CACCAT	rrnB T1; rpsI term	5' AGGAGGATGGAAAA
pYR3	In the front of the Scarlet-I3 gene	2.76; -371…-395 bp<br>-10 box TTTTATAGT Score 62<br>-35 box TTCACT Score 41	TTCACT N_{13} TATAGT	rrnB T1; rrnB T2	5' AGGAGGAAAAACAT
	In the front of the SYPF2	2.57; -560…-587 bp<br>-10 box GGCCACCAT Score 25<br>-35 box TTGAAA Score 60	TTGAAA N_{16} CACCAT	T4 term(+), T4 term(-)	5' AGGAGGATGGAAAA
pRY4	In the front of the Scarlet-I3 gene	2.76; -371…-395 bp<br>-10 box TTTTATAGT Score 62<br>-35 box TTCACT Score 41	TTCACT N_{13} TATAGT	T4 term(+), T4 term(-)	5' AGGAGGAAAAACAT
	In the front of the SYPF2	2.57; -560…-587 bp<br>-10 box GGCCACCAT Score 25	TTGAAA N_{16} CACCAT	rrnB T1; rrnB T2	5' AGGAGGATGGAAAA

		-35 box TTGAAA Score 60			

1 Only the plasmid reporter gene cassette, which contained transcription terminators flanking the cassette, reporter genes, and the region between them, was analyzed.

2 Hypothetical promoters that could activate the transcription of a given gene have been shown.

3 Hypothetical promoter sequence, with the promoter element most likely to cause promoter malfunction in bold.

4 Terminators are shown to terminate transcription from a plasmid that could cause transcription of a given gene. The terminators are located downstream of the opposing reporter gene.
