# Supplementary material for: A Reporter System for Assessment of Transcription from Divergently Oriented Promoters in Pseudomonas putida
Source: ACS Synth Biol. 2025 Dec 10;14(12):4947–66. doi: 10.1021/acssynbio.5c00723 (PMC12723746; doi:10.1021/acssynbio.5c00723)
Supplement: Supplementary file 4 [file sb5c00723_si_004.pdf]

Supporting Information for Publication

A reporter system for assessment of transcription from divergently oriented promoters in *Pseudomonas putida*

Johanna Hendrikson, Mia-Lota Keskküla, Gea M. Räis, Maia Kivisaar and Riho Teras*

Institute of Molecular and Cell Biology, University of Tartu, 51010 Estonia

* Corresponding author, riho.teras@ut.ee

Table S3. Used and constructed plasmids

Plasmid	Description/construction	Purpose/source of DNA elements	DNA element; source of DNA element; (i) Oligonucleotides used for amplification (ii) endonucleases used for the cutting of a DNA fragment	Sequence of oligonucleotide	Reference
pPR9TT	A low copy number promoter probe vector (RK2 *oriV*) containing *lacZ* reporter gene (Amp^r)	Used plasmid backbone as an RK2-type low-copy-number plasmid for the construction of reporter systems; assessment of plasmid persistence in cells			[1]

pBLKT	A medium copy number plasmid (BBR1 *oriV*) containing *lacZ* reporter gene (Km^r)	Used plasmid backbone as a BBR1-type medium-copy-number plasmid for the construction of reporter systems			[2]
pSEVA132	A medium copy number plasmid (BBR1 *oriV*, Amp^r)	Assessment of plasmid persistence in cells, a source of *bla* gene			[3]
pSEVA225	A low copy number promoter probe vector (RK2 *oriV*) containing *lacZ* reporter gene (Km^r)	Assessment of plasmid burden for cells			[3]
pSEVA235	A medium copy number plasmid (BBR1 *oriV*) containing *lacZ* reporter gene (Km^r)	Assessment of plasmid burden for cells			[3]
pBBR1MCS-2	A medium copy number plasmid (BBR1 *oriV*) containing *lacZ* reporter gene (Km^r)	Assessment of plasmid burden for cells			[4]
pKT240-Km-lacZ	A derivative of a low copy number plasmid pKT240 (RH1010 *oriV*) containing *lacZ* reporter gene (Km^r)	Assessment of plasmid burden for cells			Unpublished vector of our laboratory
pAG032	A sensor plasmid with RK2 *oriV* (Amp^r)	A source of mCerulean and mVenud genes and *hok-sok* toxin-antitoxin system			[5]
pSNW2	A promoter probe vector with R6K *oriV* (Km^r)	A source of the GFPmut2 gene			[6]
pSC101-Timer-PtetA-BFP-noDeg	An SC101 *oriV* plasmid (Km^r)	A source of the TagBFP gene			[7]
p9_PnuoA12-F3mut	RK2 origin-based promoter probe vector, containing the promoter area of *nuoA* gene with mutated Fis-nuo3 site (Cm^r, Amp^r)	A source for the *nuoA* promoter area			[8]

pBLKT_gfp-cfp	pBLKT containing divergently orientated mCerulean-GFPmut2 gene cassette with RBSs (Km^r)	mCerulean gene from pAG032 [5] and GFPmut2 gene from pSNW2 [6]	1) Vector; pBLKT;<br>(i) PCR; gfp-V2-4 and cfp-V1-2<br><br>2) gene of mCerulean; pAG032 [5];<br>(i) PCR; gfp-cfp and V1-cfp<br><br>3) gene of GFPmut2; pSNW2 [6];<br>(i) PCR; cfp-gfp4 and V2-gfp4	PCR amplified DNA fragments and plasmid was ligated by the fusion-PCR method<br>gfp-V2-4 - ACTCTACAAATAAGTATCGGCGGAATTCCAGCT<br>cfp-V1-2 - ATAATAATAAATCTACGCAAAAAGGCCATCCGTCAG<br><br>gfp-cfp - CATGCGTGGATCCATAGCAAATTAGGAGGAGGAAAATGAGCAA<br>V1-cfp - AGATCCACTAGCGATCAAGTCTTCGCGATG<br><br>cfp-gfp4 TTTGCTATGGATCCACGCATGTTAGGAGGAAAAACATATGCGTAAAGGTGAAGAACTGTTC<br>V2-gfp4 CGCCGATACTTATTTGTAGAGTTCATCCATGCCG	This study
pBLKT_cfp-yfp	pBLKT containing divergently orientated mCerulean-mVenus gene cassette with RBSs (Km^r)	mCerulean gene and mVenus gene from pAG032 [5]	1) Vector; pBLKT;<br>(i) PCR; Yfp-V2 and cfp-V1-2<br><br>2) gene of mCerulean; pAG032 [5];<br>(i) PCR; Yfp-cfp and V1-cfp-2<br><br>3) gene of mVenus; pAG032 [5];<br>(i) PCR; Cfp-yfp and V2-yfp	PCR amplified DNA fragments and plasmid was ligated by the fusion-PCR method<br>Yfp-V2 - CCGCCACCA CTGCCCG TA TTTCGCGTAAG<br>cfp-V1-2 - ATAATAATAAATCTACGCAAAAAGGCCATCCGTCAG<br><br>Yfp-cfp - AAACATGCGTGGATCCATAGCAAATAAGGAGGAGGAAAATGAGC<br>V1-cfp-2 – CCTTTTTGCGTAGATTTATTATTATTTATACAGCTCATCCATGCCAT<br><br>Cfp-yfp – GCTATGGATCCACGCATGTTTAGGAGGAAAAACATATGAGCAAAGGTGAAGAACTG<br>V2-yfp - TACGGGCAGTGGTGGCGGCTACGACAGGTAAAA	This study

pBLKT_bfp-yfp	pBLKT containing divergently orientated TagBFP-mVenus gene cassette with RBSs (Km^r)	mVenus gene from pAG032 [5] and TagBFP gene from pSC101-Timer-PtetA-BFP-noDeg [7]	1) Vector; pBLKT_cfp-yfp<br>(i) PCR; Yfp5' and bfp-V1<br><br>2) gene of TagBFP; pSC101-Timer-PtetA-BFP-noDeg [7];<br>(i) PCR; V1-bfp and yfp-bfp	PCR amplified DNA fragments and plasmid was ligated by the fusion-PCR method<br>Yfp5' – GCTATGGATCCACGCATGTTT<br>bfp-V1 – TTCGCAACGTTCAAATCCGCTC<br><br>V1-bfp - AGCGGATTTGAACGTTGCGAAG<br>yfp-bfp - AAACATGCGTGGATCCATAGCTAAGAAGGAGGTATACATATGAGTGAAGAAC	This study
pB_VS	pBLKT containing divergently orientated mVenus-Scarlet-I gene cassette with RBSs (Km^r)	The mVenus-Sacrlet-I cassette is stDNA (mV-mS(termita)), a preserved amino acid sequence with a modified DNA sequence to reduce the possibility of inner potential promoters.	1) Vector; pBLKT_bfp-yfp;<br>(i) PCR; Nhe-Acc-V5 and Sac-Xba-V6<br>(ii) Acc65I and XbaI<br><br>2) genes of mVenus and Scarlet-I; stDNA mV-mS(termita)<br>(i) Acc65I and XbaI	The DNA fragment was amplified by PCR, cut by endonucleases and ligated<br><br>Nhe-Acc-V5 - AAAGCTAGCAAGGTACCGGCGGATGATGGGGTTCTGCTTCG<br>Sac-Xba-V6 – AAAGAGCTCAAATCTAGAGCTGTCCTGCGGCCGCGCAAAAG1)	This study
pB_VS2(T)	pBLKT containing divergently orientated mVenus-Scarlet-I gene cassette with RBSs (Km^r)	Sequence of mVenus gene from pAG032 [5] and Sequence of Scarlet-I gene from pMRE145 [9]	1) Vector; pBLKT_bfp-yfp;<br>(i) PCR; Nhe-Acc-V5 and Sac-Xba-V6<br>(ii) Acc65I and XbaI<br><br>2) genes of mVenus and Scarlet-I; stDNA VS2-termita<br>(i) Acc65I and XbaI	The DNA fragment was amplified by PCR, cut by endonucleases and ligated<br><br>Nhe-Acc-V5 - AAAGCTAGCAAGGTACCGGCGGATGATGGGGTTCTGCTTCG<br>Sac-Xba-V6 - AAAGAGCTCAAATCTAGAGCTGTCCTGCGGCCGCGCAAAAG	This study
pB_VS2(R)	pBLKT containing divergently orientated mVenus-Scarlet-I gene cassette without RBSs (Km^r)	Sequence of mVenus gene from pAG032 [5] and Sequence of Scarlet-I gene from pMRE145 [9]	1) Vector; pBLKT_bfp-yfp;<br>(i) PCR; Nhe-Acc-V5 and Sac-Xba-V6<br>(ii) Acc65I and XbaI<br><br>2) genes of mVenus and Scarlet-I; stDNA VS2-RBSta<br>(i) Acc65I and XbaI	The DNA fragment was amplified by PCR, cut by endonucleases and ligated<br><br>Nhe-Acc-V5 - AAAGCTAGCAAGGTACCGGCGGATGATGGGGTTCTGCTTCG<br>Sac-Xba-V6 - AAAGAGCTCAAATCTAGAGCTGTCCTGCGGCCGCGCAAAAG	This study

pB_VS2(T)-HS	A derivative of pBLKT_VS2(T) containing toxin-antitoxin system *hok-sok* (Km^r)	The toxin-antitoxin system *hok-sok* from pAG032 [5]	1) Vector; pB_BS2(T);<br>(i) Eco32I<br><br>2) *hok-sok*; pAG032 [5];<br>(i) PCR; Hok-DraPst and Sok-PstDra<br>(ii) DraI	The DNA fragment was amplified by PCR, cut by endonucleases and ligated<br><br>Hok-DraPst – ATCCTGCAGTTTAAATTCCCACAACATCAGCAAGG<br>Sok-PstDra – GCTTTTAAACTGCAGCCGAACAAACTCCGGGAGGC	This study
pB_VS2(T)-HS-amp	A derivative of pB_VS2(T)-HS containing toxin-antitoxin system *hok-sok* (Amp^r)	The toxin-antitoxin system *hok-sok* from pAG032 [5]	1) Vector; pB_VS2(T)-HS;<br>(i) HindIII and XhoI<br><br>2) *bla* gene; pSEVA132 [3];<br>(i) PCR; PSEVA-bla1_XhoI and PSEVA-bla2_HindIII<br>(ii) HindIII and XhoI	The DNA fragment was amplified by PCR, cut by endonucleases and ligated<br><br>PSEVA-bla1_XhoI – AAACTCGAGCCAACACCCGCTGAACTAGTC<br>PSEVA-bla2_HindIII – AAAAAGCTTCACCAAGGAAAGTCTACACGAACC	This study,<br>GenBank accession number: PV786261
pB_VS2-7(T)-HS-amp	A derivative of pBLKT_VS2(T)-HS containing mVenus-ASV2-Scarlet-I-AAV gene cassette (Amp^r)	The reporter protein genes have a protein half-life-shortening tag "AAV" or "ASV2" [10]	1) Vector; pA11;<br>(i) Acc65I and XbaI<br><br>2) genes of mVenus-ASV2 and Scarlet-I-AAV; stDNA VS2-AAV-ASV2(T)<br>(i) Acc65I and XbaI	DNA fragments cut by endonucleases and ligated	This study,<br>GenBank accession number: PV786262
pPR9TT-HS	A derivative of pPR9TT containing toxin-antitoxin system *hok-sok* (Amp^r)	The toxin-antitoxin system *hok-sok* from pAG032 [5]	1) Vector; pPR9TT<br>(i) Eco105I<br><br>2) *hok-sokI*; pAG032 [5];<br>(i) PCR; Hok-DraPst and Sok-PstDra<br>(ii) DraI	The DNA fragment was amplified by PCR, cut by endonucleases and ligated<br><br>Hok-DraPst – ATCCTGCAGTTTAAATTCCCACAACATCAGCAAGG<br>Sok-PstDra – GCTTTTAAACTGCAGCCGAACAAACTCCGGGAGGC	This study
pA1	Modified pBLKT3 (BBR1, Amp^r) carrying divergently orientated reporter cassette SYFP2_Scarlet-I3, and	The medium-copy-number plasmid reporter system has been designed to study transcription in two directions simultaneously. The lack of RBSs in front of reporter genes allows the	1) Vector; pB_VS2(T)-HS-amp;<br>(i) Acc65I and XbaI	DNA fragments cut by endonucleases and ligated	This study,<br>GenBank accession number: PV786246

	toxin-antitoxin system *hok-sok*	fusion of proteins with reporter protein and gene product under study. Allows for assessing the effect of translation initiation and excludon on gene expression. The SYFP2_Scarlet-I3 cassette is stDNA, a preserved amino acid sequence with a modified DNA sequence to reduce the possibility of inner potential promoters.	2) Genes of SYFP2 and Scarlet-I3; stDNA syfp-rfp-i3(R)		
pA3	Modified pBLKT3 (BBR1, Amp^r) carrying divergently orientated reporter cassette SYFP2_Scarlet-I3 with RBS, and toxin-antitoxin system *hok-sok*	The medium-copy-number plasmid reporter system has been designed to study transcription in two directions simultaneously. The presence of RBSs in front of reporter genes enables the assessment of the effects of transcriptional regulators on gene expression. The SYFP2_Scarlet-I3 cassette is stDNA, a preserved amino acid sequence with a modified DNA sequence to reduce the possibility of inner potential promoters.	1) Vector; pB_VS2(T)-HS-amp;<br>(i) Acc65I and XbaI<br><br>2) Genes of SYFP2 and Scarlet-I3; stDNA syfp-rfp-i3	DNA fragments cut by endonucleases and ligated	This study,<br>GenBank accession number: PV786247
pA5	pBLKT3 (BBR1, Amp^r); SYFP2-ASV2_Scarlet-I3-AAV, *hok-sok*	In the pA1 variant, the reporter protein genes have a protein half-life-shortening tags "AAV" or "ASV2" [10]	1) Vector; pB_VS2(T)-HS-amp;<br>(i) Acc65I and XbaI<br><br>2) Genes of SYFP2-ASV2 and Scarlet-I3-AAV; stDNA syfp-rfp-i3-AAV-ASV2(R)	DNA fragments cut by endonucleases and ligated	This study,<br>GenBank accession number: PV786248
pA7	pBLKT3 (BBR1, Amp^r); SYFP2-ASV2_Scarlet-I3-AAV, RBS, *hok-sok*	In the pA3 variant, the reporter protein genes have a protein half-life-shortening tag "AAV" or "ASV2" [10]	1) Vector; pB_VS2(T)-HS-amp;<br>(i) Acc65I and XbaI<br><br>2) Genes of SYFP2-ASV2 and Scarlet-I3-AAV; stDNA syfp-rfp-i3-AAV-ASV2	DNA fragments cut by endonucleases and ligated	This study,<br>GenBank accession number: PV786249
pA9	pBLKT3 (BBR1, Amp^r); SYFP2-LVA2_Scarlet-I3-LVA, *hok-sok*	In the pA1 variant, the reporter protein genes have a protein half-life-shortening tag "LVA" or "LVA2" with a	1) Vector; pB_VS2(T)-HS-amp;<br>(i) Acc65I and XbaI	DNA fragments cut by endonucleases and ligated	This study,<br>GenBank accession

		modified DNA sequence coding “LVA” [10]	2) Genes of SYFP2-LVA2 and Scarlet-I3-LVA; stDNA syfp-rfp-i3-LVA(R)		number: PV786251
pA11	pBLKT3 (BBR1, Amp^r); SYFP2-LVA2_Scarlet-I3-LVA, RBS, *hok-sok*	In the pA3 variant, the reporter protein genes have a protein half-life-shortening tag "LVA" or “LVA2” with a modified DNA sequence coding “LVA” [10]	1) Vector; pB_VS2(T)-HS-amp;<br>(i) Acc65I and XbaI<br><br>2) Genes of SYFP2-LVA2 and Scarlet-I3-LVA; stDNA syfp-rfp-i3-LVA	DNA fragments cut by endonucleases and ligated	This study,<br>GenBank accession number:<br>PV786252
pB2	pBLKT3 (BBR1, Amp^r); Scarlet-I3_SYFP2, *hok-sok*	A variant of pA1 with the oppositely orientated reporter cassette	1) Vector; pB_VS2(T)-HS-amp;<br>(i) Acc65I and XbaI<br><br>2) Genes of SYFP2 and Scarlet-I3; stDNA syfp-rfp-i3(R)	DNA fragments cut by endonucleases and ligated	This study,<br>GenBank accession number:<br>PV786250
pB4	pBLKT3 (BBR1, Amp^r); Scarlet-I3_SYFP2 RBS, *hok-sok*	A variant of pA3 with the oppositely orientated reporter cassette.	1) Vector; pB_VS2(T)-HS-amp;<br>(i) Acc65I and XbaI<br><br>2) Genes of SYFP2 and Scarlet-I3; stDNA syfp-rfp-i3	DNA fragments cut by endonucleases and ligated	This study,<br>GenBank accession number:<br>PV784126
pB6	pBLKT3 (BBR1, Amp^r); Scarlet-I3-AAV_SYFP2-ASV2, *hok-sok*	In the pB2 variant, the reporter protein genes have a protein half-life-shortening tag "AAV" or “ASV2” [10]	1) Vector; pB_VS2(T)-HS-amp;<br>(i) Acc65I and XbaI<br><br>2) Genes of SYFP2-ASV2 and Scarlet-I3-AAV; stDNA syfp-rfp-i3-AAV-ASV2(R)	DNA fragments cut by endonucleases and ligated	This study,<br>GenBank accession number:<br>PV784127
pB8	pBLKT3 (BBR1, Amp^r); Scarlet-I3-AAV_SYFP2-ASV2, RBS, *hok-sok*	In the pB4 variant, the reporter protein genes have a protein half-life-shortening tag "AAV" or “ASV2” [10]	1) Vector; pB_VS2(T)-HS-amp;<br>(i) Acc65I and XbaI	DNA fragments cut by endonucleases and ligated	This study,<br>GenBank accession number:<br>PV784128

			2) Genes of SYFP2-ASV2 and Scarlet-I3-AAV; stDNA syfp-rfp-i3-AAV-ASV2		
pB10	pBLKT3 (BBR1, Amp^r); Scarlet-I3-LVA_SYFP2-LVA2, *hok-sok*	In the pB2 variant, the reporter protein genes have a protein half-life-shortening tag "LVA" or "LVA2" with a modified DNA sequence coding "LVA" [10]	1) Vector; pB_VS2(T)-HS-amp;<br>(i) Acc65I and XbaI<br><br>2) Genes of SYFP2-LVA2 and Scarlet-I3-LVA; stDNA syfp-rfp-i3-LVA(R)	DNA fragments cut by endonucleases and ligated	This study,<br>GenBank accession number: PV784129
pB12	pBLKT3 (BBR1, Amp^r); Scarlet-I3-LVA_SYFP2-LVA2, RBS, *hok-sok*	In the pB4 variant, the reporter protein genes have a protein half-life-shortening tag "LVA" or "LVA2" with a modified DNA sequence coding "LVA" [10]	1) Vector; pB_VS2(T)-HS-amp;<br>(i) Acc65I and XbaI<br><br>2) Genes of SYFP2-LVA2 and Scarlet-I3-LVA; stDNA syfp-rfp-i3-LVA	DNA fragments cut by endonucleases and ligated	This study,<br>GenBank accession number: PV784130
pYR1	Modified pPR9TT (RK2, Amp^r) carrying divergently orientated reporter cassette SYFP2_Scarlet-I3, and toxin-antitoxin system *hok-sok*	The low-copy-number plasmid reporter system has been designed to study transcription in two directions simultaneously. An analogue to pA1.	1) Vector; pPR9TT-HS;<br>(i) Acc65I and XbaI<br><br>2) Genes of SYFP2 and Scarlet-I3; stDNA syfp-rfp-i3(R)	DNA fragments cut by endonucleases and ligated	This study,<br>GenBank accession number: PV786253
pYR3	Modified pPR9TT (RK2, Amp^r) carrying divergently orientated reporter cassette SYFP2_Scarlet-I3 with RBS, and toxin-antitoxin system *hok-sok*	The low-copy-number plasmid reporter system has been designed to study transcription in two directions simultaneously. An analogue to pA3.	1) Vector; pPR9TT-HS;<br>(i) Acc65I and XbaI<br><br>2) Genes of SYFP2 and Scarlet-I3; stDNA syfp-rfp-i3	DNA fragments cut by endonucleases and ligated	This study,<br>GenBank accession number: PV786254
pYR5	pPR9TT (RK2, Amp^r); SYFP2-ASV_Scarlet-I3-AAV, *hok-sok*	In the pYR1 variant, the reporter protein genes have a protein half-life-shortening tag "AAV" or "ASV2" [10]	1) Vector; pPR9TT-HS;<br>(i) Acc65I and XbaI<br><br>2) Genes of SYFP2-ASV2 and Scarlet-I3-AAV; stDNA syfp-rfp-i3-AAV-ASV2(R)	DNA fragments cut by endonucleases and ligated	This study,<br>GenBank accession number: PV786255

pYR7	pPR9TT (RK2, Amp^r); SYFP2-ASV_Scarlet-I3-AAV, RBS, *hok-sok*	In the pYR3 variant, the reporter protein genes have a protein half-life-shortening tag "AAV" or "ASV2" [10]	1) Vector; pPR9TT-HS; (i) Acc65I and XbaI<br>2) Genes of SYFP2-ASV2 and Scarlet-I3-AAV; stDNA syfp-rfp-i3-AAV-ASV2	DNA fragments cut by endonucleases and ligated	This study, GenBank accession number: PV786256
pYR9	pPR9TT (RK2, Amp^r); SYFP2-LVA2_Scarlet-I3-LVA, *hok-sok*	In the pYR1 variant, the reporter protein genes have a protein half-life-shortening tag "LVA" or "LVA2" with a modified DNA sequence coding "LVA" [10]	1) Vector; pPR9TT-HS; (i) Acc65I and XbaI<br>2) Genes of SYFP2-LVA2 and Scarlet-I3-LVA; stDNA syfp-rfp-i3-LVA(R)	DNA fragments cut by endonucleases and ligated	This study, GenBank accession number: PV786257
pYR11	pPR9TT (RK2, Amp^r); SYFP2-LVA2_Scarlet-I3-LVA, RBS, *hok-sok*	In the pYR3 variant, the reporter protein genes have a protein half-life-shortening tag "LVA" or "LVA2" with a modified DNA sequence coding "LVA" [10]	1) Vector; pPR9TT-HS; (i) Acc65I and XbaI<br>2) Genes of SYFP2-LVA2 and Scarlet-I3-LVA; stDNA syfp-rfp-i3-LVA	DNA fragments cut by endonucleases and ligated	This study, GenBank accession number: PV786258
pRY2	pPR9TT (RK2, Amp^r); Scarlet-I3_SYFP2, *hok-sok*	A variant of pYR1 with the oppositely orientated reporter cassette	1) Vector; pPR9TT-HS; (i) Acc65I and XbaI<br>2) Genes of SYFP2 and Scarlet-I3; stDNA syfp-rfp-i3(R)	DNA fragments cut by endonucleases and ligated	This study, GenBank accession number: PV786259
pRY4	pPR9TT (RK2, Amp^r); Scarlet-I3_SYFP2 RBS, *hok-sok*	A variant of pYR3 with the oppositely orientated reporter cassette.	1) Vector; pPR9TT-HS; (i) Acc65I and XbaI<br>2) Genes of SYFP2 and Scarlet-I3; stDNA syfp-rfp-i3	DNA fragments cut by endonucleases and ligated	This study, GenBank accession number: PV784131
pRY6	pPR9TT (RK2, Amp^r); Scarlet-I3-AAV_SYFP2-ASV, *hok-sok*	In the pRY2 variant, the reporter protein genes have a protein half-life-shortening tag "AAV" or "ASV2" [10]	1) Vector; pPR9TT-HS; (i) Acc65I and XbaI<br>2) Genes of SYFP2-ASV2 and Scarlet-I3-AAV; stDNA syfp-rfp-i3-AAV-ASV2(R)	DNA fragments cut by endonucleases and ligated	This study, GenBank accession number: PV784132

pRY8	pPR9TT (RK2, Amp^r); Scarlet-I3-AAV_SYFP2-ASV, RBS, *hok-sok*	In the pRY4 variant, the reporter protein genes have a protein half-life-shortening tag "AAV" or “ASV2” [10]	1) Vector; pPR9TT-HS;<br>(i) Acc65I and XbaI<br><br>2) Genes of SYFP2-ASV2 and Scarlet-I3-AAV; stDNA syfp-rfp-i3-AAV-ASV2	DNA fragments cut by endonucleases and ligated	This study,<br>GenBank accession number:<br>PV784133
pRY10	pPR9TT (RK2, Amp^r); Scarlet-I3-LVA_SYFP2-LVA2, *hok-sok*	In the pRY2 variant, the reporter protein genes have a protein half-life-shortening tag "LVA" or “LVA2” with a modified DNA sequence coding “LVA” [10]	1) Vector; pPR9TT-HS;<br>(i) Acc65I and XbaI<br><br>2) Genes of SYFP2-LVA2 and Scarlet-I3-LVA; stDNA syfp-rfp-i3-LVA(R)	DNA fragments cut by an endonuclease and ligated	This study,<br>GenBank accession number:<br>PV784134
pRY12	pPR9TT (RK2, Amp^r); Scarlet-I3-LVA_SYFP2-LVA2, RBS, *hok-sok*	In the pRY4 variant, the reporter protein genes have a protein half-life-shortening tag "LVA" or “LVA2” with a modified DNA sequence coding “LVA” [10]	1) Vector; pPR9TT-HS;<br>(i) Acc65I and XbaI<br><br>2) Genes of SYFP2-LVA2 and Scarlet-I3-LVA; stDNA syfp-rfp-i3-LVA	DNA fragments cut by an endonuclease and ligated	This study,<br>GenBank accession number:<br>PV784135
pA3-nuoA-R<br>pA7-nuoA-R<br>pA11-nuoA-R<br>pB4-nuoA-R<br>pB8-nuoA-R<br>pB12-nuoA-R<br>pYR3-nuoA-R<br>pRY4-nuoA-R	The reporter plasmid carries a 248 bp long *nuoA* promoter region in the direction of the Scarlet-I3 gene; the promoter region contains promoter PnuoA-1, Fis binding sites Fis-nuo1, Fis-nuo2 and mutated Fis-nuo3	Reporter system validation.	1) Respective vector<br>(i) BamHI<br><br>2) *nuoA* promoter region; p9_PnuoA12-F3mut [8]<br>(i) PCR; PP4119-fw and PP4119-4-rev<br>(ii) BamHI	The DNA fragment was amplified by PCR, cut by an endonuclease and ligated<br><br>PP4119-fw – AAAGGATCCAATCGCCAGATGAACTTTAC<br>PP4119-4-rev – AAAGGATCCTCGCGCTGATTTTATGGGTA	This study
pA3-nuoA-Y<br>pA7-nuoA-Y<br>pA11-nuoA-Y<br>pB4-nuoA-Y<br>pB8-nuoA-Y<br>pB12-nuoA-Y<br>pYR3-nuoA-Y<br>pRY4-nuoA-Y	The reporter plasmid carries a 248 bp long *nuoA* promoter region in the direction of the SYFP2 gene; the promoter region contains promoter PnuoA-1, Fis binding sites Fis-nuo1, and Fis-nuo2	Reporter system validation.	1) Respective vector<br>(i) BamHI<br><br>2) *nuoA* promoter region; p9_PnuoA12-F3mut [8]<br>(i) PCR; PP4119-fw and PP4119-4-rev<br>(ii) BamHI	The DNA fragment was amplified by PCR, cut by an endonuclease and ligated<br><br>PP4119-fw – AAAGGATCCAATCGCCAGATGAACTTTAC<br>PP4119-4-rev – AAAGGATCCTCGCGCTGATTTTATGGGTA	This study

pA3-lapF-R<br>pA7-lapF-R<br>pYR3-lapF-R<br>pYR7-lapF-R	The reporter plasmid carries a 171 bp long *lapF* promoter region in the direction of the Scarlet-I3 gene; the promoter region contains promoter PlapF-1, Fis binding sites Fis-F2	Reporter system validation.	1) Respective vector<br>(i) BamHI<br><br>2) *lapF* promoter region; chromosome of *P. putida* PaW85<br>(i) PCR; LapF-fw and PP0804-rev<br>(ii) BamHI and BglII	The DNA fragment was amplified by PCR, cut by endonucleases and ligated<br><br>LapF-fw – TAGATCTTTCGCTGAGGCTTTTCTAC<br>PP0804-rev – TGGATCCACTTCGGATTGCTTATCGG	This study
pA3-lapF-Y<br>pA7-lapF-Y<br>pYR3-lapF-Y<br>pYR7-lapF-Y	The reporter plasmid carries a 171 bp long *lapF* promoter region in the direction of the SYFP2 gene; the promoter region contains promoter PlapF-1, Fis binding sites Fis-F2	Reporter system validation.	1) Respective vector<br>(i) BamHI<br><br>2) *lapF* promoter region; chromosome of *P. putida* PaW85<br>(i) PCR; LapF-fw and PP0804-rev<br>(ii) BamHI and BglII	The DNA fragment was amplified by PCR, cut by endonucleases and ligated<br><br>LapF-fw – TAGATCTTTCGCTGAGGCTTTTCTAC<br>PP0804-rev – TGGATCCACTTCGGATTGCTTATCGG	This study
pBVS2-7-L-AY-FR	The derivative of reporter plasmid pB_VS2-7(T(-HS) carrying a 419 bp long fusion promoter area of outwardly directed *nuoA* and *lapF* promoter areas. The promoter PnuoA-1 is in front of SYFP2, and P-lapF-1 is in front of the Scarlet-I3 gene.	Reporter system validation.	1) The respective vector<br>(i) BamHI<br><br>2) promoter area of *lapF*; pRY8-lapF-R<br>(i) PCR; nuoA-lapF and lapF-revII<br><br>3) promoter area of *nuoA*; pA7-nuoA-Y<br>(i) PP4119-4-rev and lap-FnuoA<br><br>4) generation of artificial *nuoA-lapF* fusion promoter area by fusion PCR<br>(i) BamHI	The fusion promoter area was generated by fusion-PCR of amplified PCR fragments of promoter areas, and the DNA was cut by an endonuclease and ligated<br><br>nuoA-lapF – CATCTGGCGATTTCGCTGAGGCTTTTCTACCAAAG<br>lapF-revII – AAAGATCCCCCGGCCCGAGTGTTCGGG<br><br>PP4119-4-rev – AAAGGATCCTCGCGCTGATTTTATGGGTA<br>lapFnuoA – CCTCAGCGAAATCGCCAGATGAACTTTACAAGG	This study

pA3_L_AR_FY<br>pA7_L_AR_FY	The reporter plasmid (pA3 or pA7) carries a 419 bp long fusion promoter area of outwardly directed *nuoA* and *lapF* promoter areas. The promoter PnuoA-1 is in front of Scarlet-I3, and P-lapF-1 is in front of the SYFP2 gene.	Reporter system validation.	1) The respective vector<br>(i) BamHI<br><br>2) promoter area of *lapF*; pRY8-lapF-R<br>(i) PCR; nuoA-lapF and lapF-revII<br><br>3) promoter area of *nuoA*; pA7-nuoA-Y<br>(i) PP4119-4-rev and lap-FnuoA<br><br>4) generation of artificial *nuoA-lapF* fusion promoter area by fusion PCR<br>(i) BamHI	The fusion promoter area was generated by fusion-PCR of amplified PCR fragments of promoter areas, and the DNA was cut by an endonuclease and ligated<br><br>nuoA-lapF – CATCTGGCGATTTCGCTGAGGCTTTTCTACCAAAG<br>lapF-revII – AAAGATCCCCCGGCCCGAGTGTTCGGG<br><br>PP4119-4-rev – AAAGGATCCTCGCGCTGATTTTATGGGTA<br>lapFnuoA – CCTCAGCGAAATCGCCAGATGAACTTTACAAGG	This study
pA3_L_AY_FR<br>pA7_L_AY_FR	The reporter plasmid (pA3 or pA7) carrying a 419 bp long fusion promoter area of outwardly directed *nuoA* and *lapF* promoter areas. The promoter PnuoA-1 is in front of SYFP2, and P-lapF-1 is in front of the Scarlet-I3 gene.	Reporter system validation.	1) The respective vector<br>(i) BamHI<br><br>2) promoter area of *lapF*; pRY8-lapF-R<br>(i) PCR; nuoA-lapF and lapF-revII<br><br>3) promoter area of *nuoA*; pA7-nuoA-Y<br>(i) PP4119-4-rev and lap-FnuoA<br><br>4) generation of artificial *nuoA-lapF* fusion promoter area by fusion PCR<br>(i) BamHI	The fusion promoter area was generated by fusion-PCR of amplified PCR fragments of promoter areas, and the DNA was cut by an endonuclease and ligated<br><br>nuoA-lapF – CATCTGGCGATTTCGCTGAGGCTTTTCTACCAAAG<br>lapF-revII – AAAGATCCCCCGGCCCGAGTGTTCGGG<br><br>PP4119-4-rev – AAAGGATCCTCGCGCTGATTTTATGGGTA<br>lapFnuoA – CCTCAGCGAAATCGCCAGATGAACTTTACAAGG	This study
pC13A-AAV	A derivative of pPR9TT-HS, containing DNA	Synthetically combined DNA cassette containing:	1) vector; pPR9TT-HS<br>(i) Acc65I and XbaI	DNA fragments cut by endonucleases and ligated	This study,

	cassette rrnB T1T2-*lacI*^{q}-TL17-Ptac-LacIoperator-Scarlet-I3-AAV-Pm-RNAI-*xylS*-T4-T4 (Amp^r)	(i) transcriptional terminators rrnB T1T2 and T4 from pPR9TT$_B$lacZ [11], TL17 and RNAI from pAG032 [5]<br>Promoters: Ptac (mRNA of Scarlet-I3), Pm (antisense strand for mRNA of Scarlet-I3)<br>Genes: *lacI*^{q} from pBRlacItac [12], *xylS* from pAG032 [5], Scarlet-I3-AAV from pA7.<br>Reporter system validation.	2) reporter cassette; stDNA<br>(i) Acc65I and XbaI		GenBank accession number: PV786260

1. Santos, P. M.; Blatny, J. M.; Di Bartolo, I.; Valla, S.; Zennaro, E., Physiological analysis of the expression of the styrene degradation gene cluster in *Pseudomonas fluorescens* ST. *Applied and environmental microbiology* **2000,** *66* (4), 1305-10.
2. Lahesaare, A.; Moor, H.; Kivisaar, M.; Teras, R., *Pseudomonas putida* Fis binds to the *lapF* promoter in vitro and represses the expression of LapF. *PloS one* **2014,** *9* (12), e115901.
3. Silva-Rocha, R.; Martinez-Garcia, E.; Calles, B.; Chavarria, M.; Arce-Rodriguez, A.; de Las Heras, A.; Paez-Espino, A. D.; Durante-Rodriguez, G.; Kim, J.; Nikel, P. I.; Platero, R.; de Lorenzo, V., The Standard European Vector Architecture (SEVA): a coherent platform for the analysis and deployment of complex prokaryotic phenotypes. *Nucleic acids research* **2013,** *41* (Database issue), D666-75.
4. Hao, L.; Liu, X.; Wang, H.; Lin, J.; Pang, X.; Lin, J., Detection and validation of a small broad-host-range plasmid pBBR1MCS-2 for use in genetic manipulation of the extremely acidophilic Acidithiobacillus sp. *Journal of microbiological methods* **2012,** *90* (3), 309-14.
5. Gawin, A.; Peebo, K.; Hans, S.; Ertesvag, H.; Irla, M.; Neubauer, P.; Brautaset, T., Construction and characterization of broad-host-range reporter plasmid suitable for on-line analysis of bacterial host responses related to recombinant protein production. *Microbial cell factories* **2019,** *18* (1), 80.
6. Wirth, N. T.; Kozaeva, E.; Nikel, P. I., Accelerated genome engineering of Pseudomonas putida by I-SceI-mediated recombination and CRISPR-Cas9 counterselection. *Microbial biotechnology* **2020,** *13* (1), 233-249.
7. Boothe, T.; Lim, G. E.; Cen, H.; Skovso, S.; Piske, M.; Li, S. N.; Nabi, I. R.; Gilon, P.; Johnson, J. D., Inter-domain tagging implicates caveolin-1 in insulin receptor trafficking and Erk signaling bias in pancreatic beta-cells. *Mol Metab* **2016,** *5* (5), 366-378.
8. Teppo, A.; Lahesaare, A.; Ainelo, H.; Samuel, K.; Kivisaar, M.; Teras, R., Colonization efficiency of *Pseudomonas putida* is influenced by Fis-controlled transcription of *nuoA-N* operon. *PloS one* **2018,** *13* (8), e0201841.
9. Schlechter, R. O.; Jun, H.; Bernach, M.; Oso, S.; Boyd, E.; Munoz-Lintz, D. A.; Dobson, R. C. J.; Remus, D. M.; Remus-Emsermann, M. N. P., Chromatic Bacteria - A Broad Host-Range Plasmid and Chromosomal Insertion Toolbox for Fluorescent Protein Expression in Bacteria. *Frontiers in microbiology* **2018,** *9*, 3052.
10. Andersen, J. B.; Sternberg, C.; Poulsen, L. K.; Bjorn, S. P.; Givskov, M.; Molin, S., New unstable variants of green fluorescent protein for studies of transient gene expression in bacteria. *Applied and environmental microbiology* **1998,** *64* (6), 2240-6.
11. Kivistik, P. A.; Putrins, M.; Puvi, K.; Ilves, H.; Kivisaar, M.; Hõrak, R., The ColRS two-component system regulates membrane functions and protects *Pseudomonas putida* against phenol. *Journal of bacteriology* **2006,** *188* (23), 8109-17.
12. Ojangu, E. L.; Tover, A.; Teras, R.; Kivisaar, M., Effects of combination of different -10 hexamers and downstream sequences on stationary-phase-specific sigma factor sigma(S)-dependent transcription in *Pseudomonas putida*. *Journal of bacteriology* **2000,** *182* (23), 6707-13.
