# Supplementary material for: A Reporter System for Assessment of Transcription from Divergently Oriented Promoters in Pseudomonas putida
Source: ACS Synth Biol. 2025 Dec 10;14(12):4947–66. doi: 10.1021/acssynbio.5c00723 (PMC12723746; doi:10.1021/acssynbio.5c00723)
Supplement: Supplementary file 5 [file sb5c00723_si_005.pdf]

Supporting Information for Publication

A reporter system for assessment of transcription from divergently oriented promoters in *Pseudomonas putida*

Johanna Hendrikson, Mia-Lota Keskküla, Gea M. Räis, Maia Kivisaar and Riho Teras*

Institute of Molecular and Cell Biology, University of Tartu, 51010 Estonia

* Corresponding author, riho.teras@ut.ee

Table S4. Used oligonucleotides for RT-qPCR.

Gene	Oligonucleotide	Sequence of oligonucleotide	Reference
Gene expression dependency on reporter cassette orientation experiments			
Scarlet-I3	qPCR-S13-fw	5' CGTCCGTATGAAGGCACC	This study
	qPCR-S13-rev	5' AGAATATCCCAGCTAAACGGC	This study
SYFP2	qPCR-SRY2-fw	5' GCAAATCAGTTTCAGGGTCAG	This study
	qPCR-SRY2-rev	5' CCATAAGTTTAGCGTGTCGGG	This study
rpoD	rpoD-fw	5' CGATGGAAATCACCAGAC	This study
	rpoD-rev	5' GCTGATCGACCTTGAGAC	This study
Excludon mechanism studies			
cDNA from the sense strand (mRNA)	I-s-SI3	5' AAGCTCTGTTTCCAATAATCCG	This study
cDNA from the antisense strand (asRNA)		5' GAAATTGAAGGCGAAGGTGAAG	This study
amplification	qPCR-S13-fw	5' CGTCCGTATGAAGGCACC	This study
	qPCR-S13-rev	5' AGAATATCCCAGCTAAACGGC	This study
polA	polAXhoylev	5' GGG GCA GAA CGC CAA GTA CG	[1]
	polAXhoall	5' TCT GCG CCA GGC TGT CCA T	[1]

1. Sidorenko, J.; Jatsenko, T.; Saumaa, S.; Teras, R.; Tark-Dame, M.; Horak, R.; Kivisaar, M., Involvement of specialized DNA polymerases Pol II, Pol IV and DnaE2 in DNA replication in the absence of Pol I in Pseudomonas putida. *Mutation research* **2011,** *714* (1-2), 63-77.
